# Pathogenicity of *Cadophora luteo-olivacea* on *Quercus robur* and multi-omics characterization of antagonism by *Trichoderma atroviride*

**DOI:** 10.1038/s41598-026-59030-8

**Published:** 2026-06-22

**Authors:** Lucie Frejlichová, Miroslav Berka, Martin Černý, Dana Homolová, David Gramaje, Aleš Eichmeier

**Affiliations:** 1 Faculty of Horticulture, Mendeleum - Institute of Genetics, Mendel University in Brno, Brno, Czech Republic; 2 Department of Forest Protection and Wildlife Management, Faculty of Forestry and Wood Technology, Mendel University in Brno, Brno, Czech Republic; 3 Department of Molecular Biology and Radiobiology, Faculty of AgriSciences, Mendel University in Brno, Brno, Czech Republic; 4 Instituto de Ciencias de la Vid y del Vino (ICVV), Spanish National Research Council (CSIC), University of La Rioja and Government of La Rioja, 26007 Logroño, Spain

**Keywords:** Oak, *Cadophora luteo-olivacea*, *Trichoderma atroviride*, Koch’s postulates, Mycoparasitism, Proteomics, Microbiology, Plant sciences

## Abstract

Pedunculate oak (*Quercus robur* L.) is a foundation tree species in European forests and reforestation programs, but nursery propagated seedlings can harbor cryptic trunk diseases pathogens. *Cadophora luteo-olivacea*, known from grapevine trunk diseases, has been detected in oak nurseries, yet its pathogenicity on oak and interactions with antagonistic fungi remain unclear. We fulfilled Koch’s postulates for *C. luteo-olivacea* isolate CZ_395 on *Q. robur* seedlings under experimental inoculation conditions and quantified growth reduction of *C. luteo-olivacea* by *Trichoderma atroviride* isolate CZ_180 in dual culture. Proteomic and metabolomic profiling of the contact zone was performed at two post contact sampling points, 4 and 8 dpi, to identify candidate molecular signatures associated with the interaction. Inoculated seedlings developed extensive stem lesions (mean 11.9 cm), whereas controls showed minimal wound response (mean 0.9 cm; *p* < 0.001). In dual culture, *T. atroviride* reduced the visible colony development and radial growth of *C. luteo-olivacea* under the tested in vitro conditions. Contact zone proteomics revealed 257 differentially abundant proteins at 8 days, including cell wall targeting hydrolases, secreted proteases, oxidoreductases (44 upregulated), and transporters. Metabolomics detected contact specific changes in amino acids, central carbon intermediates, and lipid-associated features, including reduced ergosterol. This study demonstrates that *C. luteo-olivacea* can induce necrotic lesions in *Q. robur* under experimental inoculation conditions and identifies proteomic and metabolomic signatures associated with the interaction between *T. atroviride* and *C. luteo-olivacea*, providing a basis for nursery risk assessment and future evaluation of biocontrol potential.

**Supplementary Information:**

The online version contains supplementary material available at https://doi.org/10.1038/s41598-026-59030-8.

## Introduction

Pedunculate oak (*Quercus robur* L.) is a keystone of European temperate forests, contributing to biodiversity maintenance, carbon storage, and timber production^1–4^. Climate change, recurrent droughts, and pest outbreaks have intensified reforestation efforts that favor broadleaf species such as *Q. robur*^5–10^. Millions of oak seedlings are produced annually in Central European nurseries^5,6^, turning nurseries into critical phytosanitary checkpoints where asymptomatic seedlings can disseminate latent fungal pathogens^11,12^. Trunk disease pathogens (TDPs) cause severe losses in forestry and agriculture^13–15^, with families such as Botryosphaeriaceae, Nectriaceae and Diaporthaceae impacting a broad range of woody hosts, including oaks^16,17^. Yet pathogen presence alone does not equate to disease; outcomes also depend on the surrounding microbiome and soil context^18–22^.

Among lesser studied fungi linked to wood decay, *Cadophora luteo-olivacea* (J.F.H. Beyma) T.C. Harr. & McNew, 2003 (Leotiomycetes, Helotiales, Ploettnerulaceae) is repeatedly detected in grapevine nurseries and vineyards and has also been reported from fruit crops such as kiwifruit and apple, where selected strains have shown pathogenic potential^23–29^. Its regular detection in asymptomatic grapevine nursery material and vineyard soils underscores a risk of cryptic dissemination^25,29^. However, to our knowledge, Koch’s postulates have not yet been fulfilled for *C. luteo-olivacea* on *Q. robur*, and molecular level information on its interactions with antagonistic fungi remains limited.

*Trichoderma* spp. are well established biocontrol fungi and plant beneficial endophytes that suppress pathogens through a combination of mycoparasitism, antibiosis, resource competition, and modulation of host defenses^30–32^. Across diverse confrontation systems, secretome/proteome studies repeatedly implicate cell wall degrading enzymes (e.g., chitinases and β-glucanases), proteases, redox/detox enzymes, transporters, and small secreted proteins as key components of the antagonistic repertoire^33–36^. However, how these responses unfold in space and time particularly at the physical interaction front with trunk disease associated fungi remains insufficiently resolved. Metabolomics can complement proteome/secretome profiling by revealing small molecule signals and antifungal compounds that underpin antagonism and plant priming^32,37–39^.

Here, we examined two linked components of a nursery associated pathosystem: the interaction between *Q. robur* and the trunk disease associated fungus *C. luteo-olivacea* in an experimental inoculation assay, and the in vitro interaction between *C. luteo-olivacea* and a natural isolate of *T. atroviride* (CZ_180) with putative antagonistic potential. High-throughput amplicon sequencing of field collected samples^40^ detects *Trichoderma* and *Cadophora* in oak nursery stock, but whether their co-occurrence reflects antagonism, neutral coexistence, or context-dependent associations remains unresolved. Here, we addressed two linked experimental questions: (i) whether *C. luteo-olivacea* isolate CZ_395 can induce necrotic lesions in *Q. robur* seedlings under experimental inoculation conditions and be re-isolated from symptomatic tissue, and (ii) how *T. atroviride* CZ_180 affects the in vitro colony growth of *C. luteo-olivacea* CZ_395 in dual culture. In addition, proteomic and metabolomic profiling of the Trichoderma contact zone at two post contact time points, 4 and 8 dpi, was used to identify candidate proteins, metabolites, and pathways associated with the antagonistic interaction. Together, these complementary assays connect nursery detection patterns with experimental pathogenicity testing and molecular signatures associated with the antagonistic interaction, providing a basis for future evaluation of biocontrol potential in a foundation forest species^1,30,39^.

## Results

### Pathogenicity of *Cadophora luteo-olivacea* on *Q. robur*

**Fig. 1:**
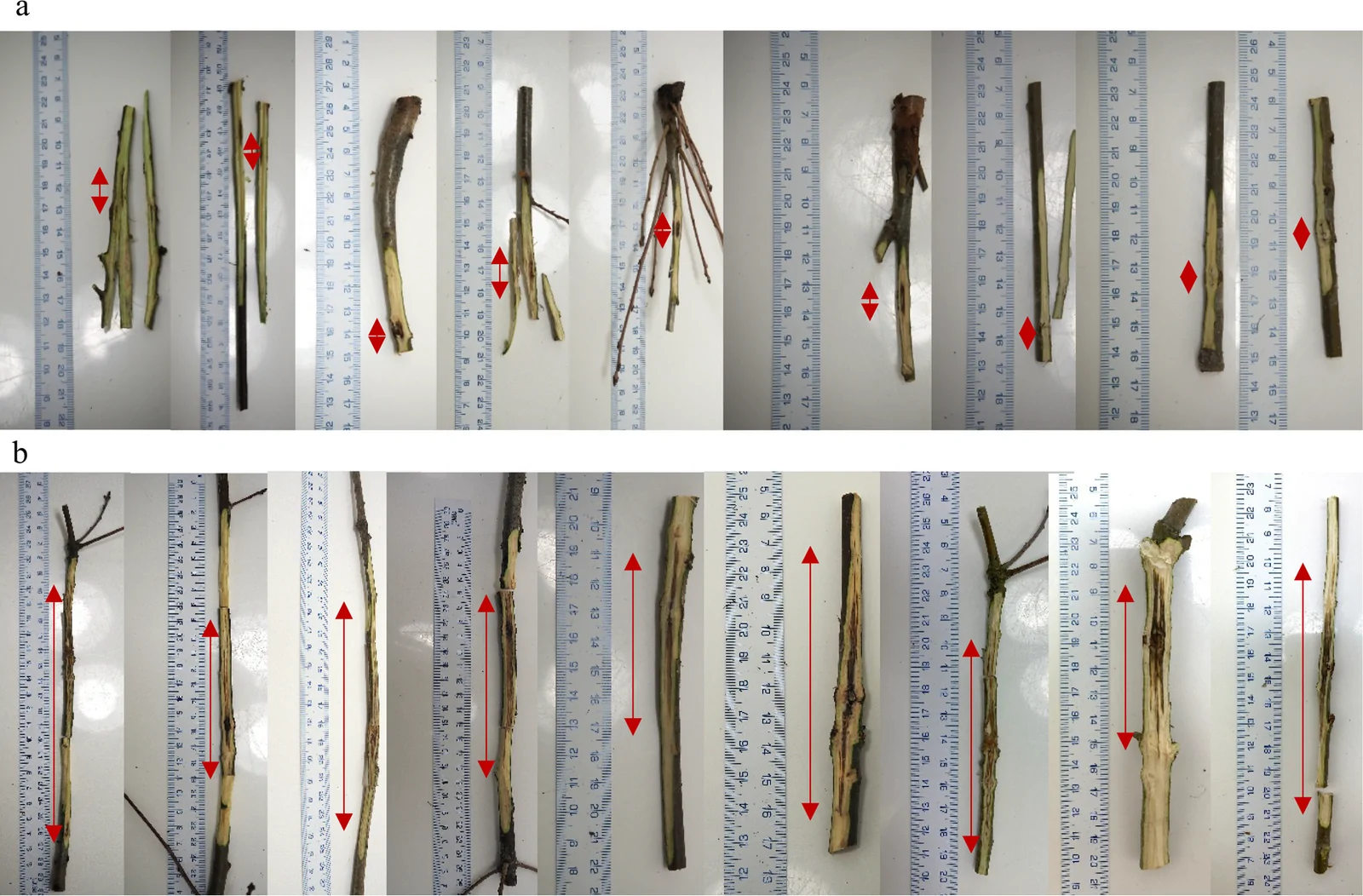
Representative stem symptoms in control and *C. luteo-olivacea*-inoculated *Q. robur* seedlings. (**a**) Mock inoculated control seedlings showing limited wound associated discoloration. (**b**) Seedlings inoculated with *C. luteo-olivacea* isolate CZ_395 showing more extensive stem discoloration and necrotic lesion development. Red arrows indicate the measured symptomatic area used for lesion assessment.

Inoculated *Q. robur* seedlings developed substantially longer stem lesions than controls (Fig. 1; Supplementary Tables S2–S5). Mean lesion length reached 11.88 ± 6.27 cm in *C. luteo-olivacea* inoculated seedlings, compared with 0.72 ± 0.58 cm in controls. A linear model using lesion length as the dependent variable, inoculation treatment as the main explanatory variable, and plant length as a covariate confirmed a significant effect of inoculation treatment. Inoculated seedlings had lesions estimated to be 11.47 cm longer than controls (*p* = 3.79 × 10^−6^), whereas plant length itself was not a significant predictor of lesion length (*p* = 0.222).

Minor wound associated discoloration was observed in some controls, but it remained limited in extent and *C. luteo-olivacea* was not recovered from any control plant. In contrast, *C. luteo-olivacea* was consistently re-isolated from symptomatic inoculated seedlings (21/21), and multilocus sequencing (ITS, TEF1-α, RPB2) confirmed that the re-isolated strains corresponded to *C. luteo-olivacea* isolate CZ_395 (NCBI Acc.: PX843264–PX843267, PX898111–PX898114, PX898115–PX898119). Together, lesion development after inoculation, absence of the target fungus from controls, and successful re-isolation from inoculated seedlings support fulfilment of Koch’s postulates under the tested experimental inoculation conditions (Fig. 1).

### Dual culture interaction assay

**Fig. 2:**
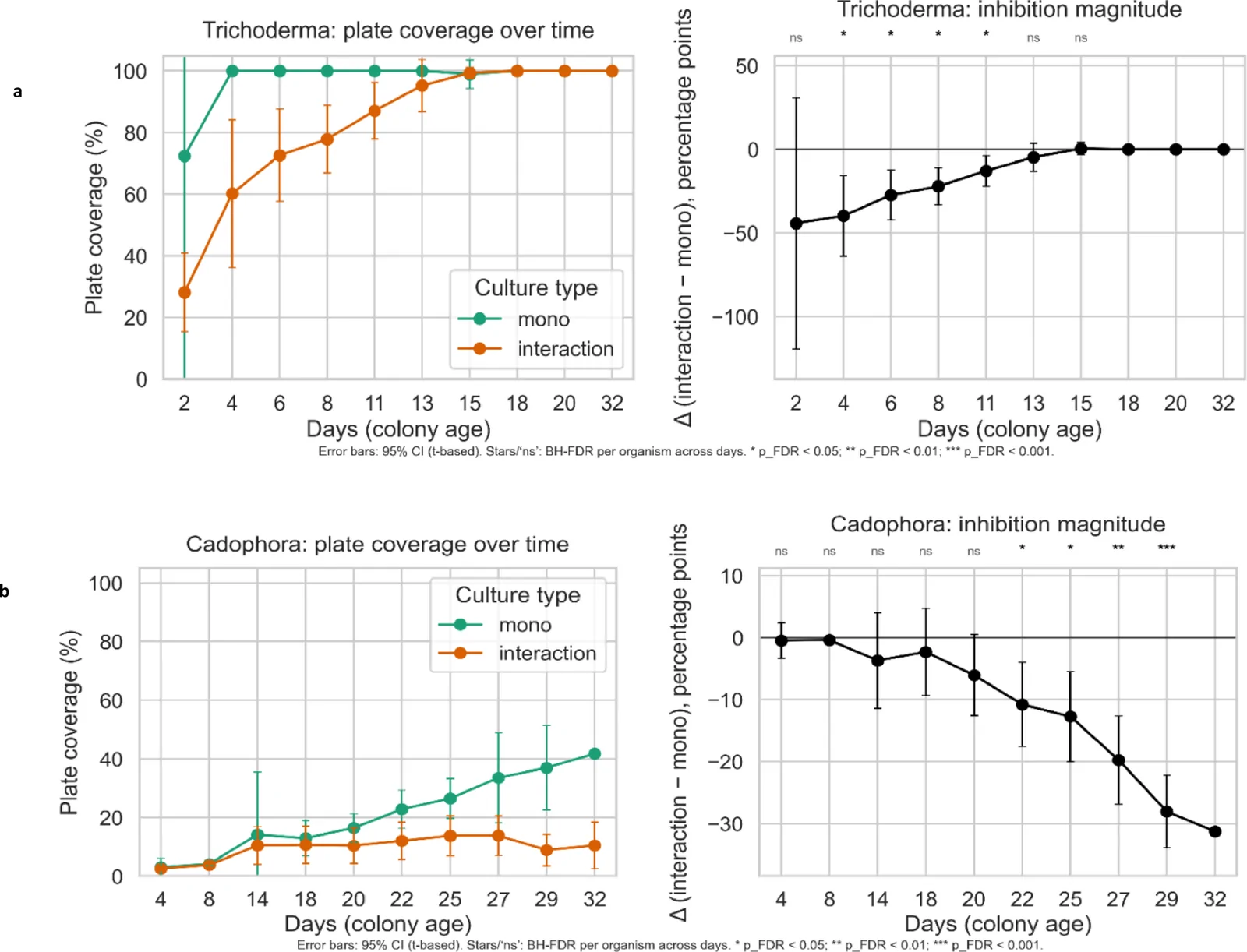
Visible colony development in dual culture. (**a**) Plate coverage of *C. luteo-olivacea* CZ_395 and *T. atroviride* CZ_180 in monoculture and dual culture over time (means ± 95% CI). *C. luteo-olivacea* was pregrown for 14 d before *T. atroviride* inoculation. (**b**) Difference in visible plate coverage expressed as Δ = dual culture − monoculture in percentage points. Negative values indicate lower visible plate coverage in dual culture than in monoculture. For *C. luteo-olivacea*, negative Δ values indicate reduced visible colony area in the presence of *T. atroviride* under the tested assay geometry. *T. atroviride* coverage in dual culture was interpreted cautiously because visual assignment was limited to distinguishable mycelium or sporulating growth on the plate surface. Significance per day is BH-FDR adjusted across sampling days within each organism and measured variable.

In vitro dual culture assays showed that *T. atroviride* CZ_180 (NCBI Acc.: PX842629, PX927109) reduced the visible colony area of *C. luteo-olivacea* CZ_395 (NCBI Acc.: PX842612, PX898121, PX898120). In monoculture, *C. luteo-olivacea* expanded slowly, reaching approximately 35–40% plate coverage at the final sampling points, whereas the visible colony area attributable to *C. luteo-olivacea* remained below 20% in dual culture with *T. atroviride* (Fig. 2a and b; Supplementary Table S6). *T. atroviride* rapidly colonized the plates and reached full coverage within 10–12 days in monoculture. In dual culture, the visible plate coverage attributable to *T. atroviride* was lower during early time points and approached full coverage as the interaction progressed (Fig. 2a and b).

For each organism, visible plate coverage in monoculture and dual culture was compared separately at each sampling day using Welch’s two sample t-test, with Benjamini–Hochberg (BH) correction applied across sampling days within each organism (Fig. 2a and b; Supplementary Table S7). The coverage difference, expressed as Δ = dual culture—monoculture in percentage points, was used as a descriptive measure of visible plate coverage changes between monoculture and dual culture. Negative Δ values indicated lower visible plate coverage in dual culture than in monoculture. For *C. luteo-olivacea*, negative Δ values indicated reduced visible colony area under dual culture conditions with *T. atroviride*. Macroscopic observations showed overgrowth of *C. luteo-olivacea* colonies by *T. atroviride* in dual culture (Supplementary Fig. S2).

#### Global proteomic response of *Trichoderma atroviride*

De novo peptide sequences (PEAKS 6.0) were taxonomically profiled using Unipept to verify organism attribution across monocultures and cocultures (Supplementary Fig. S2 and S3). Monoculture samples showed genus consistent peptide profiles. In dual culture, samples collected from the *C. luteo-olivacea* side of the contact zone contained peptides assigned to both *C. luteo-olivacea* and *T. atroviride*, whereas samples collected from the *T. atroviride* side did not show a corresponding *C. luteo-olivacea* peptide contribution. This pattern is consistent with physical overgrowth and colonization of the *C. luteo-olivacea* colony by *T. atroviride* in the confrontation area, resulting in mixed species material on the *Cadophora* side of the contact zone. Therefore, downstream organism specific proteome profiling was restricted to samples collected from the *T. atroviride* side of the contact zone. Samples collected from the *C. luteo-olivacea* side of the contact zone were excluded from organism specific proteome interpretation because they represented mixed species material with ambiguous protein assignment.

**Fig. 3:**
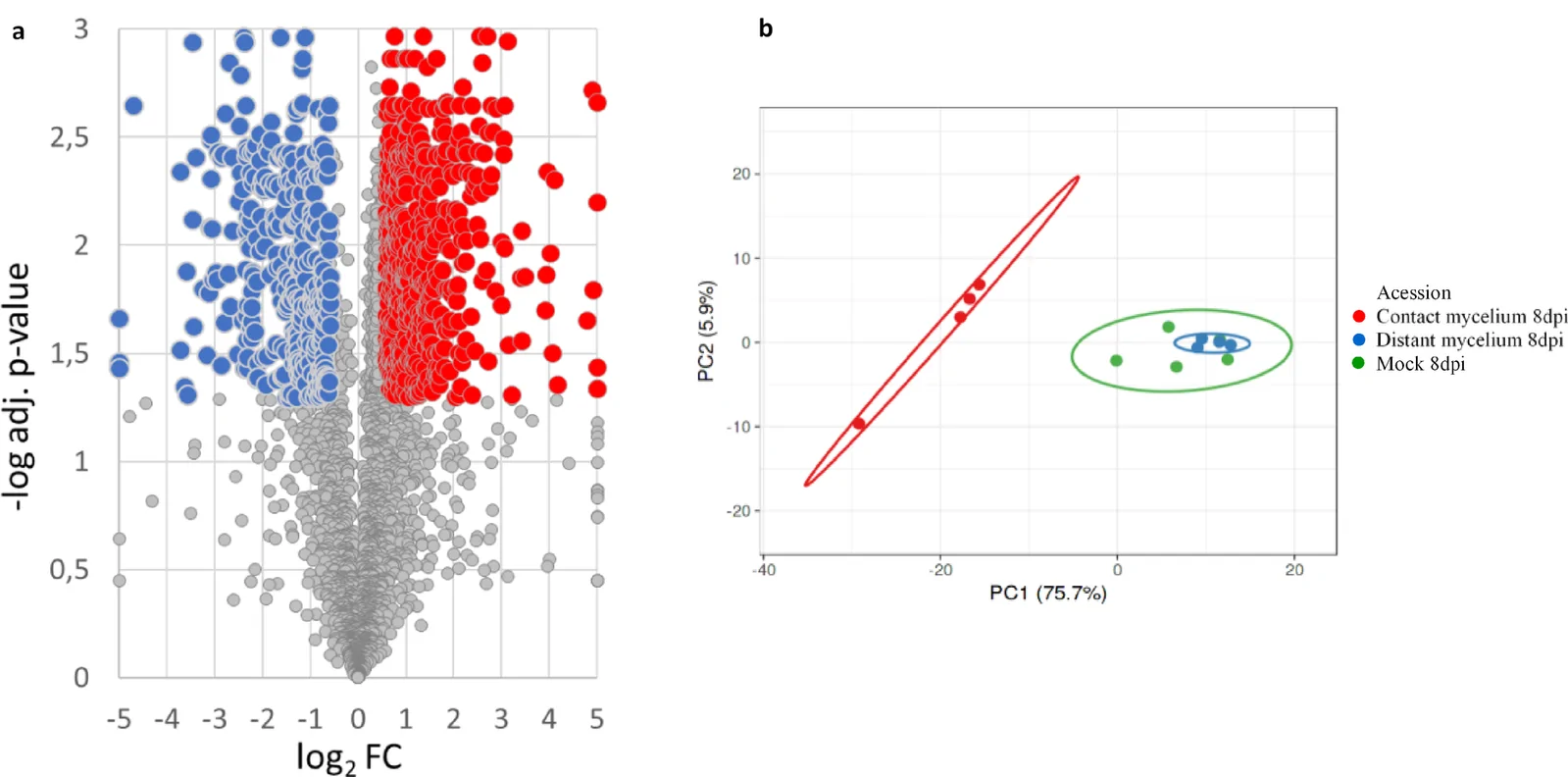
Global proteomic response of *T. atroviride* at 8 dpi. (**a**) Volcano plot using FDR < 0.05 and |log_2_FC|≥ 0.58 (~ 1.5×); red = up, blue = down, grey = nonsignificant, highlighting strong contact associated shifts. (**b**) PCA of replicate samples separates the contact zone (8 dpi) from mock/distal (MOCK 8 dpi/INT 8 dpi) primarily along PC1 (75.7% variance.

A total of 1060 proteins were quantified (Supplementary Tables S31 and S32). At 8 dpi, differentially abundant proteins (DAPs) were defined using predefined criteria combining statistical significance and effect size (FDR < 0.05 and |log_2_FC|≥ 0.58, corresponding to a ≥ 1.5-fold change). This analysis revealed substantial changes in the quantified proteome at the *Trichoderma* side of the interaction front. Contact versus mock (cvm) yielded 257 DAPs (235 up, 22 down) and contact versus distant (cvd) yielded 244 DAPs (222 up, 22 down) (Fig. 3a). Most DAPs were shared between contrasts (216 upregulated and 21 downregulated), indicating that the proteomic response was concentrated in the contact zone rather than being equally reflected in distant *T. atroviride* mycelium (Supplementary Fig. S4). Consistently, PCA separated contact samples from mock and distant samples primarily along the first principal component (Fig. 3b).

To contextualize these changes at a broad functional level, we compared the relative frequency of proteins assigned to broad annotation categories across conditions (Supplementary Fig. S5, Supplementary Table S9). Oxidoreductases represented the most frequent annotated category across all conditions, accounting for approximately 20–23% of the quantified proteins included in this category level summary. At 8 dpi, contact zone samples showed a lower relative representation of RNA/ribosome associated proteins and a higher proportion of unannotated proteins compared with mock and distant samples. This pattern indicates that the contact zone proteome included a larger fraction of proteins without functional annotation and a reduced representation of translation associated categories, rather than indicating a fully resolved functional shift.

#### Carbohydrate active enzymes (CAZymes)

**Fig. 4:**
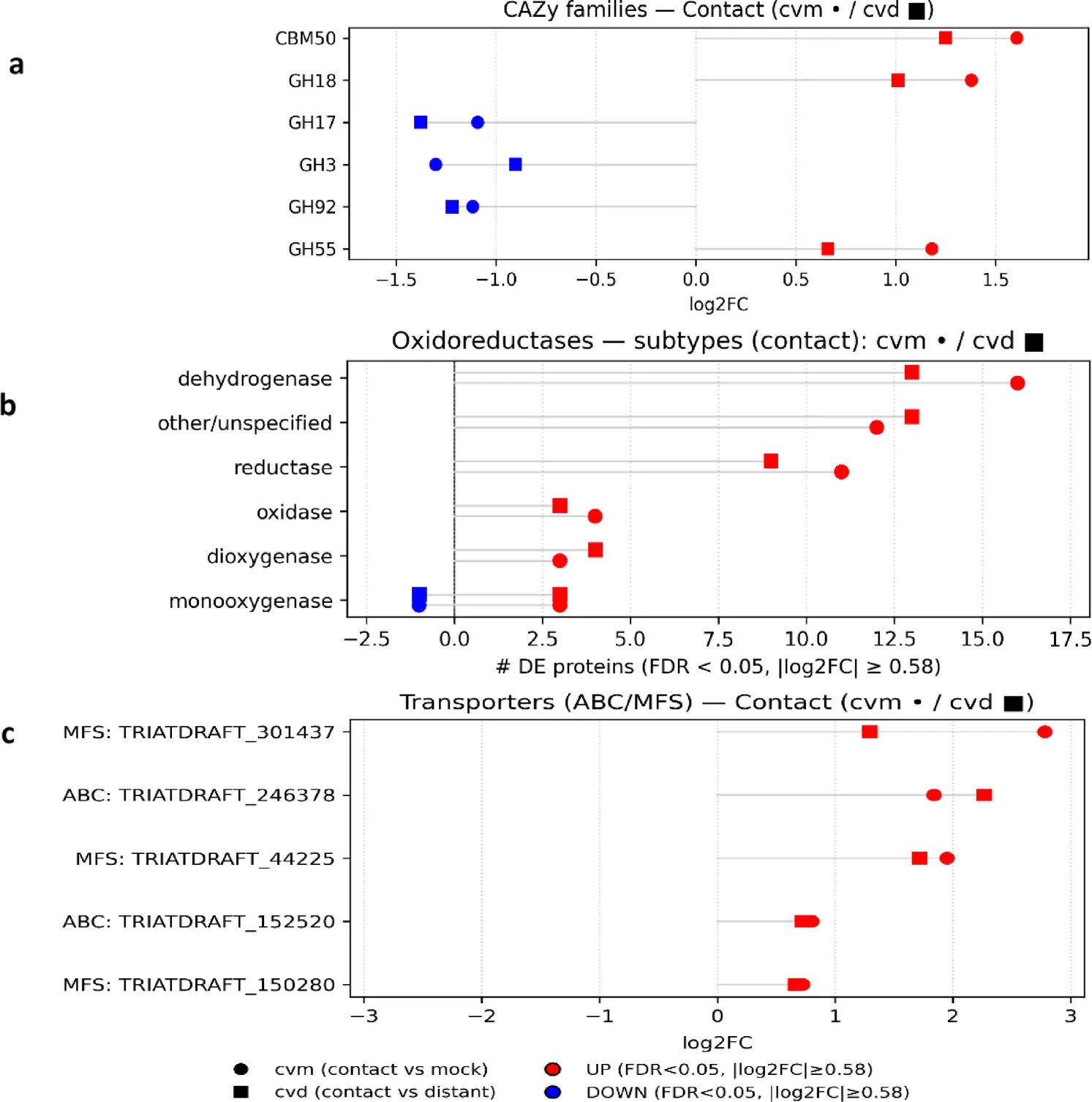
Contact induced changes in CAZy families, oxidoreductases and transporters. (**a**) Differentially abundant CAZy families in contact zone mycelium shown as log_2_FC relative to mock (cvm, circles) and distant (cvd, squares) (Supplementary Table S10). (**b**) Numbers of differentially abundant oxidoreductases per subtype in the contact zone for cvm and cvd (Supplementary Table S11). (**c**) Selected ABC and MFS transporters significantly changed in the contact zone (log_2_FC for cvm and cvd) (Supplementary Table S12).

At 8 dpi, CAZy proteins were evaluated using predefined DAP criteria combining statistical significance and effect size (FDR < 0.05 and |log_2_FC|≥ 0.58, corresponding to a ≥ 1.5-fold change). The CAZy response at the *Trichoderma* side of the contact zone involved a limited set of carbohydrate active enzymes and binding modules associated with fungal cell wall related substrates (Fig. 4a; Supplementary Table S10). The GH18 chitinase G9P0A7 was upregulated in both contrasts (cvm: log_2_FC =  + 1.38, q = 0.0168; cvd: log_2_FC =  + 1.01, q = 0.0037). Additional upregulated CAZy related proteins included a CBM50/LysM protein (G9NJY3) and a GH55 exo-β-1,3-glucanase (G9NNK5). In contrast, several glycoside hydrolases were downregulated in both contrasts, including GH17 endo-β-1,3-glucanase G9P2J8 (cvm: log_2_FC = − 1.09, q = 0.012; cvd: log_2_FC = − 1.38, q = 0.0049), GH3 β-glucosidase G9P6Z3 (cvm: log_2_FC = − 1.30, q = 0.047; cvd: log_2_FC = − 0.90, q = 0.0025), and GH92 α-mannosidase G9NK86 (cvm: log_2_FC = − 1.12, q = 0.026; cvd: log_2_FC = − 1.22, q = 0.0043). Thus, the CAZy profile indicated selective changes in chitin and β-glucan associated functions rather than broad upregulation of all fungal cell wall degrading enzymes.

#### Oxidoreductases and secondary metabolism

No oxidoreductases were significantly induced at 4 dpi, whereas at 8 dpi oxidoreductase associated proteins were predominantly more abundant in contact zone samples (Fig. 4b; Supplementary Table S11). In total, 44 oxidoreductases were differentially abundant in the contact versus mock contrast (cvm; 43 upregulated, 1 downregulated), and 42 were differentially abundant in the contact versus distant contrast (cvd; 41 upregulated, 1 downregulated). The most represented subclasses among upregulated oxidoreductases were dehydrogenases (16 in cvm; 13 in cvd) and reductases (11 in cvm; 9 in cvd), followed by monooxygenases (3 in cvm; 3 in cvd), dioxygenases (3 in cvm; 4 in cvd), and oxidases (4 in cvm; 3 in cvd). Upregulated proteins included multiple FAD/FMN binding oxidoreductases, short chain dehydrogenase/reductases, 2-oxoglutarate dependent dioxygenases, an amine oxidase, an FR type ferric chelate reductase, and enzymes linked to secondary metabolism, including polyketide synthase and enoyl reductase domains. One monooxygenase, G9PC98, was consistently downregulated in both contrasts (cvm: log_2_FC = − 0.99; cvd: log_2_FC = − 2.44).

#### Transport and detoxification mechanisms

Ten ABC/MFS transporters were detected (Fig. 4c; Supplementary Table S12). Six were significantly upregulated at 8 dpi (3 in cvm and 3 in cvd), and none were significantly downregulated. The induced set comprised ABCG/PDR transporters and several MFS sugar/proton symporters, consistent with elevated metabolite flux and compound export at the interaction front.

#### Small secreted and cell wall associated proteins (non enzymatic)

Two hydrophobins (G9NTN2, G9P4V8) were significantly enriched in the 8 dpi contact zone versus mock (cvm; BH-FDR), whereas neither reached significance in the contact versus distant contrast (cvd), consistent with a predominantly contact localized signal. Cerato platanin/EPL2 (G9NTJ1) was detected but was not differentially abundant in either contrast (Supplementary Table S9). The calcofluor white hypersensitive protein (G9PBB5) was significantly enriched in contact versus mock at both 4 and 8 dpi (Supplementary Table S9).

#### GO/InterPro over representation and subcellular composition (GO:CC)

Four upregulated protein sets (4 dpi interaction vs. mock; 8 dpi contact vs. mock, cvm; 8 dpi contact vs. distant, cvd; and 8 dpi distant vs. mock, dvm) were tested for GO and InterPro enrichment using Fisher’s exact tests with BH-FDR correction (α = 0.05). Target sets were defined as proteins with log_2_FC ≥ 0.58, and the background was restricted to GO annotated proteins. After FDR correction, only one GO cellular component term, extracellular region (GO:0005576), was significantly enriched in the 4 dpi interaction versus mock comparison. At 8 dpi, two InterPro signatures were significantly enriched in the contact zone contrasts: GNAT domain (IPR000182) and Acyl-CoA N-acyltransferase domain (IPR016181) (Supplementary Fig. S6; Supplementary Table S13). No GO Biological Process or Molecular Function terms passed FDR correction.

Because only few GO cellular component terms were significantly enriched at 8 dpi, we additionally calculated abundance based Δ share values for each GO term relative to the corresponding mock condition. For each condition, the summed abundance of proteins assigned to a given GO term was expressed as a percentage of the total quantified GO proteome, and Δ share was calculated as the percentage point difference relative to mock. In the 8 dpi contact zone comparison, the largest positive Δ share was observed for cytoplasm (+ 3.82 percentage points), whereas mitochondrion (− 1.24 percentage points), nucleus (− 1.21 percentage points), and plasma membrane (− 0.92 percentage points) showed the largest negative Δ share values. The remaining GO categories showed smaller changes, with the next largest absolute Δ share reaching 0.56 percentage points. The complete abundance share values and Δ share values are provided in Supplementary Tables S14–S16 and visualized in Supplementary Figs. S7 and S8.

#### Condition dependent proteins (INT vs. MOCK)

Ranked, within condition heatmaps of log₁₀ relative abundances (Supplementary Fig. S9 and S10; Table S17–S22) show that the dominant proteome is largely shared between conditions, but hyphal contact markedly reorders which proteins dominate, highlighting shifts in proteome composition rather than simply the number of regulated proteins. Proteins were ranked by mean abundance in the reference condition (Top 30; n = 4) and tested by Welch’s t-test with BH-FDR correction (★ BH-FDR < 0.05); arrows indicate INT versus MOCK direction. Already at 4 dpi, the interaction displays an early surface focused signature, with increased interface associated proteins (e.g., hydrophobins G9NTN2/G9P4V8 and the PIR like cell wall protein G9NXA8) alongside reduced abundance of several high ranking housekeeping/metabolic proteins (e.g., enolase G9NHJ7), consistent with early metabolic reallocation during confrontation.

By 8 dpi in the contact zone, the dominance pattern shifts toward proteins associated with antagonistic activity (CAZy/peptidase/redox/transport modules highlighted above; Fig. 4; Supplementary Table S10), whereas the distant mycelium shows only weak systemic changes, in line with the limited enrichment signal at 8 dpi (Supplementary Fig. S12; Table S13). A direction of change summary based on two sided tests is provided in supplementary Table S9, and full ranked lists are given in supplementary Tables S17–S22.

#### Metabolomic response of *Trichoderma atroviride*

**Fig. 5:**
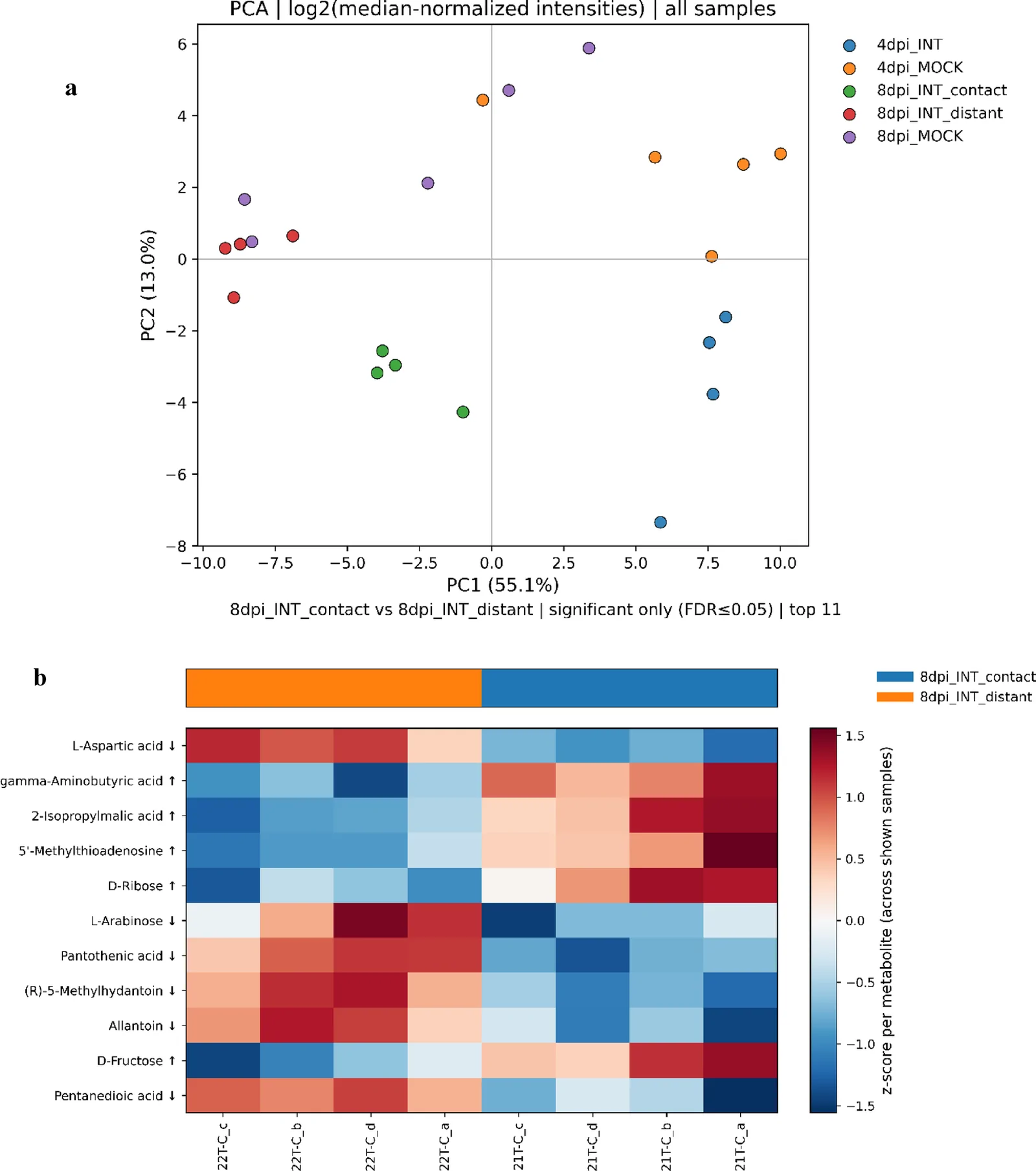
Time and contact dependent shifts in the polar metabolome during the Trichoderma-Cadophora interaction. (**a**) PCA of log_2_ median normalized polar metabolite intensities across all samples separates 4 versus 8 dpi and distinguishes 8 dpi conditions (contact, distant, mock). (**b**) Heatmap of the top 11 polar metabolites for 8 dpi contact versus distant (ranked by BH–FDR; Supplementary Table S26) shown as per metabolite z-scores.

In total, 1517 compounds were putatively annotated by GC–MS analysis combined with Compound Discoverer. Of these, 87 metabolites met the predefined identification criteria: a library match score > 80 and a retention index deviation (ΔRI) < 1% between the experimentally observed and reference retention index values. PCA of the polar fraction (log_2_ median normalized intensities; Fig. 5a; Supplementary Table S23) separated 4 dpi from 8 dpi along PC1 (55.1%) and further distinguished 8 dpi contact, distant, and mock samples along PC2 (13.0%). PERMDISP supported the lowest within group dispersion for the 8 dpi contact group (Supplementary Fig. S12; Table S25).

Consistent with a weak early response, univariate testing detected only two significant polar metabolites at 4 dpi (BH-FDR < 0.05; upregulated N-acetylornithine and downregulated trehalose in INT vs. MOCK; Supplementary Fig. S13; Table S30). In contrast, the metabolomic signal broadened at 8 dpi across contact versus mock (cvm; Supplementary Fig. S14; Table S28) and distant versus mock (dvm; Supplementary Fig. S15), and was most clearly resolved in replicate paired contact versus distant (cvd; 11 metabolites; Fig. 5b; Supplementary Table S29). Significant polar metabolites mainly comprised sugars/central carbon intermediates, amino acid related compounds, and stress/turnover or cofactor linked features, with heatmaps recapitulating contrast specific signatures at 8 dpi.

The nonpolar fraction showed a similar condition dependent shift at 8 dpi (PCA: Supplementary Fig. S11; Supplementary Fig. S16; Supplementary Table S31), including sterol associated changes in the contact zone versus mock, with decreased ergosterol and an unknown sterol (median log_2_FC = − 0.82 and − 0.83, respectively; BH-FDR ≤ 0.014), and increased 5-hydroxy-2-(hydroxymethyl)pyridine (median log_2_FC = + 0.36; BH-FDR = 0.020). These changes were also reflected in the nonpolar metabolite heatmaps.

#### Cross omic integration and mechanistic anchors

To link metabolite changes with protein regulation, we compiled a pathway level cross omics process summary that aggregates significant metabolites and differentially abundant proteins into shared process clusters and reports directionality using median log_2_ fold changes (Table 1). Metabolite significance (BH-FDR) was assessed on log_2_ (median normalized intensities) using two sided Welch’s t-tests for unpaired contrasts (INT vs. MOCK; contact vs. mock; distant vs. mock) and two sided replicate paired t-tests for paired contrasts (cvd and cvm; paired by replicate), with Benjamini Hochberg correction applied across metabolites within each contrast (q ≤ 0.05).

**Table 1:** Pathway level cross omics process clusters (metabolomics proteomics) at 4 and 8 dpi. Significant metabolites (BH–FDR q ≤ 0.05) and differentially abundant proteins were grouped into shared process clusters. Metabolites were tested on log_2_ median-normalized intensities (Welch’s t-test for unpaired contrasts; paired t-tests for cvd/cvm) with BH correction. “Met effect” and “Prot effect” are median log_2_ fold changes; “–” indicates none. Full counts: Supplementary Table S37; metabolite-protein pairs: Supplementary Table S36.

Contrast	Process cluster	Met effect	Prot effect	Representative pathways
4DPI_INT__MOCK	Amino acids & sulfur aa metabolism	Down	–	Amino acid metabolism
8DPI_CVD	Amino acids & sulfur aa metabolism	Up	Up	Amino-acid biosynthesis; L-arginine biosynthesis; L-histidine biosynthesis
8DPI_CVD	Carbohydrates & glycans	Up	Down	Carbohydrate metabolism; Carbohydrate degradation; Glycan metabolism
8DPI_CVD	Central carbon/tca/pyruvate	Mixed	–	Central carbon/TCA/pyruvate
8DPI_CVD	Cofactors & one-carbon	–	Up	Cofactor biosynthesis; FMN biosynthesis; NAD(+) biosynthesis
8DPI_CVD	Energy & salvage	–	Up	IMP biosynthesis via salvage pathway; Ketone metabolism
8DPI_CVD	Lipids & membranes	–	Down	Lipid metabolism; Phospholipid metabolism; phosphatidylethanolamine biosynthesis
8DPI_CVD	Nucleotides & purines	Up	Up	Nucleotide metabolism; Purine metabolism; XMP biosynthesis via de novo pathway
8DPI_CVD	Other	–	Up	Metabolic intermediate biosynthesis
8DPI_CVD	Protein modification/regulation	–	Up	Protein modification; protein lipoylation via endogenous pathway; protein ubiquitination
8DPI_CVD	Secondary metabolism	–	Up	Secondary metabolite biosynthesis; terpenoid biosynthesis
8DPI_CVD	Stress/redox	Up	–	Stress/redox related (putative)
8DPI_CVM	Amino acids & sulfur aa metabolism	Down	Up	Amino-acid biosynthesis; L-lysine biosynthesis via AAA pathway; Amino acid metabolism
8DPI_CVM	Carbohydrates & glycans	Up	down	Carbohydrate metabolism; Carbohydrate degradation; Glycan metabolism
8DPI_CVM	Central carbon/tca/pyruvate	Up	–	Central carbon/TCA/pyruvate
8DPI_CVM	Cofactors & one-carbon	–	Up	Cofactor biosynthesis; FMN biosynthesis; NAD(+) biosynthesis
8DPI_CVM	Energy & salvage	–	Up	IMP biosynthesis via salvage pathway; Ketone metabolism
8DPI_CVM	Lipids & membranes	–	Down	Lipid metabolism; Phospholipid metabolism; phosphatidylethanolamine biosynthesis
8DPI_CVM	Nucleotides & purines	Up	Up	Purine metabolism; Nucleotide metabolism; AMP biosynthesis via de novo pathway
8DPI_CVM	Other	–	Up	Metabolic intermediate biosynthesis; L-leucine biosynthesis
8DPI_CVM	Protein modification/regulation	–	Up	Protein modification; protein lipoylation via endogenous pathway; protein ubiquitination
8DPI_CVM	Secondary metabolism	–	Up	Secondary metabolite biosynthesis; terpenoid biosynthesis
8DPI_CVM	Stress/redox	Up	–	Stress/redox related (putative)

At 4 dpi, the cross omics signal was minimal (one significant metabolite mapped to the amino acids & sulfur amino acid metabolism cluster). In contrast, at 8 dpi the response broadened and was most pronounced in amino acid related processes. The amino acids & sulfur amino acid metabolism cluster contained 26 significant hits in contact versus distant (cvd; 2 metabolites, 24 proteins) and 34 hits in contact versus mock (cvm; 3 metabolites, 31 proteins). Across clusters, the type of cross omic support differed: some clusters showed dual support (metabolites and proteins significant), whereas several were protein driven (proteins significant with no metabolites passing BH-FDR), consistent with interaction associated remodeling of enzymatic capacity without necessarily producing detectable shifts in metabolite pools (or falling below GC–MS coverage/sensitivity).

As conservative mechanistic anchors, was additionally evaluated predefined metabolite protein pairs (Supplementary Table S36). This presence based mapping separates differential evidence from detection only coverage and highlights one sided versus dual support at specific nodes a transsulfuration node (CBS–cystathionine; CBS significant at 8 dpi, cystathionine detected but not BH-FDR significant), a methionine salvage node (MTAP–5′-methylthioadenosine; 5′-methylthioadenosine significant in cvd), and a central carbon node (enolase/pyruvate; pyruvate significant in cvm).

## Discussion

This study shows that *Cadophora luteo-olivacea* CZ_395 can induce necrotic stem lesions in *Quercus robur* seedlings under experimental inoculation conditions and can be re-isolated from symptomatic tissue, supporting fulfilment of Koch’s postulates within this artificial assay system. These findings are consistent with an opportunistic pathogenic role of *C. luteo-olivacea* in wounded or otherwise favourable host conditions, rather than demonstrating disease development under natural nursery conditions. In parallel, dual culture assays showed reduced visible colony development of *C. luteo-olivacea* in the presence of *Trichoderma atroviride* CZ_180, while proteomic and metabolomic profiling identified candidate molecular processes associated with the interaction. By combining pathogenicity testing, in vitro interaction phenotyping, and omics based profiling, this study links nursery detection of *C. luteo-olivacea* with experimentally observed lesion formation and provides a basis for further evaluation of *T. atroviride* as a potential biocontrol organism.

Inoculated seedlings developed extensive necrotic stem lesions, whereas controls showed only minimal wound response, demonstrating that *C. luteo-olivacea* CZ_395 can induce necrosis in *Q. robur* seedlings under the tested experimental wounded stem inoculation conditions. Successful re-isolation and molecular confirmation support fulfilment of Koch’s postulates within this artificial assay system, providing experimental evidence for an opportunistic pathogenic capacity of *C. luteo-olivacea* on *Q. robur*. This finding is relevant in the context of repeated detection of *C. luteo-olivacea* in asymptomatic grapevine nursery material^25^, and its association with trunk disease systems in grapevine^27,28,41^, where latent or weakly expressed infections can contribute to pathogen dissemination. Although the present study does not demonstrate natural disease development in oak nurseries, it indicates that the occurrence of *C. luteo-olivacea* in forest nursery stock should not be considered biologically irrelevant and warrants further targeted surveillance and phytosanitary evaluation^12^.

In dual culture assays, *T. atroviride* CZ_180 reduced the visible colony development and radial growth of *C. luteo-olivacea* under the tested in vitro conditions, with up to 50% inhibition of radial growth and macroscopic overgrowth of the *Cadophora* colony. This pattern is consistent with the frequently reported antagonistic behaviour of *Trichoderma* species against fungal plant pathogens^31,32,42,43^. The detection of *Trichoderma* and *Cadophora* in oak nursery stock by high-throughput amplicon sequencing^40^ indicates that these fungi can occur within the same nursery associated system, but such co-detection alone does not demonstrate antagonism or direct interaction^44–46^. The present in vitro assay and omics profiling therefore provide experimental evidence for an interaction between the selected isolates and identify candidate molecular processes associated with *T. atroviride* mycoparasitic behaviour. Further experiments under plant or nursery relevant conditions will be required to evaluate the practical biocontrol potential of *T. atroviride* against *C. luteo-olivacea* and related trunk disease-associated fungi^13,47^.

Contact zone proteomics revealed a temporally staged and spatially confined response. At 4 dpi, the interaction was characterized by an early surface focused signature, with increased abundance of interface associated proteins including hydrophobins (G9NTN2, G9P4V8) and a PIR-like cell wall mannoprotein (G9NXA8), alongside reduced abundance of several housekeeping and metabolic proteins (e.g., enolase G9NHJ7). This pattern is consistent with early metabolic reallocation and preparation of the cell surface for confrontation. By 8 dpi, the response intensified markedly, with 257 differentially abundant proteins in the contact zone (235 upregulated, 22 downregulated), dominated by enzymes targeting fungal cell walls, secreted proteases, oxidoreductases, and transporters. The spatial confinement of this response, with the contact zone showing dramatically different protein profiles compared to distant mycelium, indicates that *T. atroviride* deploys a localized, interface specific antagonistic program rather than a systemic response. This spatial specificity is consistent with direct mycoparasitism, where localized secretion of lytic enzymes and metabolites at the interaction front enables efficient resource allocation and minimizes self damage^39,43,48^.

The increased abundance of selected CAZymes associated with fungal cell wall substrates is consistent with previously described features of *Trichoderma* mycoparasitism, in which chitinases and β-glucanases contribute to recognition and degradati on of the target fungal cell wall^39,42^. In the present study, a GH18 chitinase (G9P0A7) was significantly more abundant in the contact zone, consistent with a role in chitin degradation, as chitin is a major structural component of fungal cell walls. Chitin hydrolysis may weaken the target cell wall and can also release chitin-derived oligosaccharides that have been linked to stimulation of *Trichoderma* antagonistic responses^39,42^. Interestingly, a GH17 endo-β-1,3-glucanase (G9P2J8) was downregulated, while a GH55 exo-β-1,3-glucanase (G9NNK5) was upregulated, suggesting selective changes in β-glucan associated enzymes rather than uniform induction of all fungal cell wall-degrading CAZymes. The downregulation of certain β-glucanases may reflect self protection, as *Trichoderma* cell walls also contain β-1,3-glucan^49,50^. The detection of a CBM50/LysM domain protein (G9NJY3) is consistent with a possible role in chitin binding or recognition during the interaction. This interpretation is aligned with previous reports that LysM domains bind chitin oligosaccharides and can contribute to enzyme localization at fungal cell walls or to modulation of host immune recognition^42^.

Beyond CAZymes, secreted proteases may contribute to the interaction by degrading extracellular or cell wall associated proteins of the target fungus and by modifying the local protein environment at the contact zone. The increased abundance of oxidoreductases at 8 dpi is consistent with redox-associated processes previously reported during fungal antagonism and *Trichoderma* mycoparasitism, including ROS-related stress responses and redox cycling^39,43^. However, because ROS production was not directly measured in the present study, these proteins should be interpreted as candidate indicators of redox remodeling rather than direct evidence for ROS-mediated antagonism. ROS can damage fungal cells and may also function as signaling molecules coordinating *Trichoderma* developmental and metabolic responses^39,43^. The induction of multiple transporter proteins, including ABCG/PDR-type efflux pumps and MFS transporters, is consistent with previously described roles in secondary metabolite transport, xenobiotic efflux, and detoxification during fungal interactions^31,42^.

In parallel with the increased abundance of selected fungal cell wall associated enzymes, *T. atroviride* showed enrichment of a calcofluor white hypersensitive protein (G9PBB5), suggesting changes in cell wall associated stress responses during the interaction. Calcofluor White binds fungal cell wall polysaccharides, particularly chitin, and can disturb cell wall assembly, thereby inducing compensatory cell wall stress responses in fungi^50–52^. Calcofluor white hypersensitive proteins have also been linked to stress tolerance and pathogenicity in the entomopathogenic fungus *Metarhizium acridum*^53^*.* In the present study, enrichment of G9PBB5, together with additional wall associated proteins such as the PIR-like mannoprotein G9NXA8 and β-1,3-glucan associated remodeling enzymes, is therefore consistent with a contact zone response involving cell wall stress perception or remodeling. However, direct reinforcement of the *T. atroviride* cell wall, changes in β-glucan crosslinking, or altered wall composition were not experimentally tested here.

Analysis of the nonpolar metabolite fraction revealed contact specific changes in sterol related features, with ergosterol, the principal fungal membrane sterol, significantly decreased in the interaction zone, alongside changes in a putative sterol precursor or derivative. Ergosterol is important for fungal membrane stability and ordering^54,55^. The observed sterol associated changes may therefore indicate lipid associated adaptation during confrontation related stress. However, because membrane properties were not directly measured, these results should be interpreted as candidate envelope associated responses rather than direct evidence for membrane remodeling. Together, the enrichment of cell wall associated proteins and the changes in sterol related metabolites suggest that the interaction zone involved coordinated changes in fungal cell envelope associated processes, while the functional consequences of these changes require targeted validation^50,51^.

The pronounced induction of two large multidomain polyketide synthases (G9NYD1, G9NTK1) at both 4 dpi and 8 dpi indicated sustained engagement of secondary metabolomic pathways during the interaction. Polyketide synthases drive the biosynthesis of diverse secondary metabolites including antibiotics, toxins, and pigments, which can inhibit pathogen growth and modulate the competitive outcome^31,39,56^. The concomitant downregulation of several core primary metabolic enzymes (e.g., glycolytic and basal respiration proteins) in the contact zone, suggests that carbon flux and energy are redirected from growth-related processes toward secondary metabolism^39,43^. Such metabolic reprogramming is consistent with previously described *Trichoderma* responses during fungal confrontation and competitive interactions in vitro and in soil or plant associated systems, where secondary metabolites may complement enzymatic attack^42,48^.

Although many complex polyketide products are not directly detectable by our GC–MS setup, the nonpolar metabolite profile showed unique metabolites accumulating under interaction conditions, including a distinctive pyridine derivative (annotated as 5-hydroxy-2-(hydroxymethyl)pyridine). *Trichoderma* species are widely reported to deploy secondary metabolites during competition and biocontrol^31,39,57^, and the detection of altered lipophilic compounds—including unidentified peaks in the nonpolar fraction—reinforces that small-molecule chemistry is extensively remodeled as part of the competitive response^38,58^. This coordination between proteome and metabolome highlights chemical output as a key axis of antagonism^38,39^.

A third major response axis involves the reproducible induction of a linoleate diol synthase (LDS, G9NXJ9) in the interaction zone. LDS type fatty acid oxygenases are involved in oxylipin biosynthesis, and fungal oxylipins have been implicated in the regulation of development, stress responses, and secondary metabolism^59,60^. The increased abundance of LDS therefore suggests a possible involvement of oxylipin related lipid metabolism during the confrontation. However, because oxylipins were not directly quantified in the present study, this result should be interpreted as a candidate lipid signaling related response rather than direct evidence that *T. atroviride* uses lipid derived messengers to coordinate antagonistic activity.

Free unsaturated fatty acids, including linoleic acid, accumulated to higher levels in the contact treatment compared to the mock, consistent with enhanced substrate availability for oxylipin biosynthesis^59^. The parallel reduction in ergosterol content, noted above, further indicates reorganization of membrane lipid composition^54,55^. Together, these changes paint a picture of a membrane in flux: *T. atroviride* likely recycles and modifies membrane components both to generate lipid-derived signals and to adapt membrane properties during confrontation^59,60^.

Together, the observed changes in cell wall associated proteins, secondary metabolism related enzymes, and LDS/oxylipin associated lipid metabolism suggest that the *T. atroviride* contact zone response involved several candidate processes previously linked to fungal confrontation and *Trichoderma* antagonism^39,43,51,59,60^. Rather than demonstrating a single coordinated mechanism, our data indicate that the interaction zone was associated with changes in proteins related to cell wall remodeling or stress responses, secondary metabolic capacity, and lipid derived regulatory pathways. These patterns are consistent with previously reported features of antagonistic fungal interactions, but direct functional roles, including cell wall reinforcement, inhibitory activity of specific secondary metabolites, or membrane remodeling, were not experimentally tested here^43,48,59,60^.

## Conclusions

This study demonstrates that *Cadophora luteo-olivacea* CZ_395 can induce stem lesions in wounded *Quercus robur* seedlings under experimental inoculation conditions and can be re-isolated from symptomatic tissue, supporting fulfilment of Koch’s postulates within this artificial assay system. Dual culture assays showed reduced visible colony development and radial growth of *C. luteo-olivacea* in the presence of *Trichoderma atroviride* CZ_180 under the tested in vitro conditions. Proteomic and metabolomic profiling at two post contact sampling points identified candidate molecular processes associated with the *T. atroviride* contact zone response, including CAZymes, proteases, oxidoreductases, transporters, secondary-metabolism-related enzymes, and lipid associated features. These results provide a basis for future functional validation and for evaluating *Trichoderma* isolates under plant and nursery relevant conditions.

## Materials and methods

### Fungal isolates

**Fig. 6:**
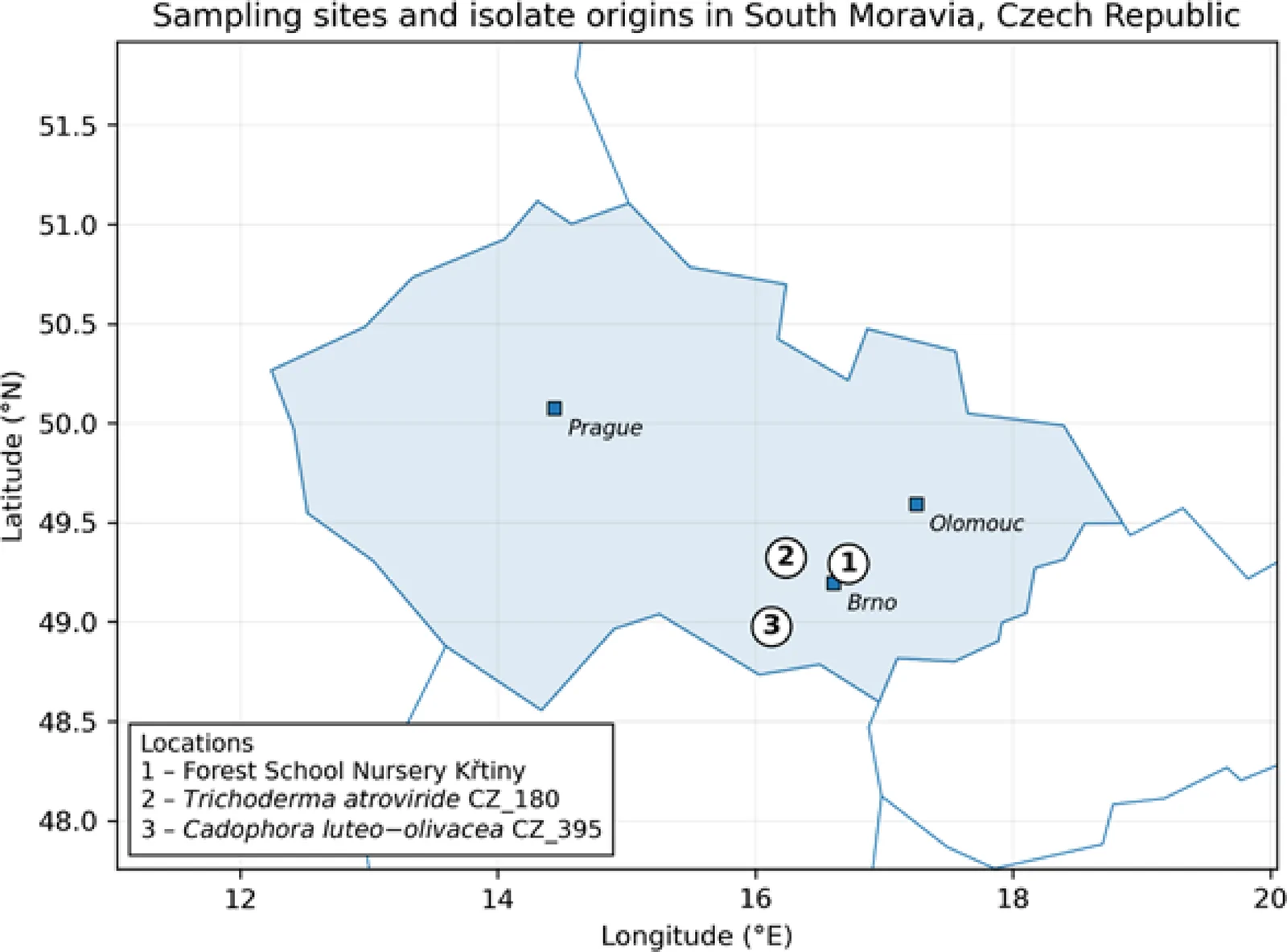
Locations of sampling sites and isolate origins in South Moravia, Czech Republic. (1) Forest School Nursery Křtiny, Q. robur seedlings used for Koch’s postulates; (2) source of the Trichoderma atroviride isolate CZ_180; (3) source of the *Cadophora luteo-olivacea* isolate CZ_395. Major cities are shown only for geographic orientation. The map was created by the authors in Python 3.13.5 using GeoPandas v1.1.2 and Matplotlib v3.10.8, with country boundary data obtained from the Natural Earth Admin 0-Countries vector dataset at 1:110 million scale (https://www.naturalearthdata.com/). Sampling points were plotted from GPS coordinates generated in this study.

Two fungal isolates were selected for pathogenicity and antagonism assays. The candidate pathogenic isolate *C. luteo-olivacea* CZ_395 and the candidate antagonistic isolate *Trichoderma atroviride* CZ_180 were obtained in spring 2021 from trunk disease (TD) symptomatic two year old bare rooted *Quercus robur* seedlings. Symptoms included internal stem discoloration, necrotic lesions, chlorosis consistent. *Cadophora luteo-olivacea* was isolated from seedlings sampled at site 3 (48.9749733°N, 16.1258161°E), and *T. atroviride* from seedlings sampled at site 2 (49.3218225°N, 16.2383442°E) (Fig. 6). The potential antagonistic role of *T. atroviride* was not inferred from its occurrence in symptomatic material, but from the known antagonistic capacity of *Trichoderma* spp. and was experimentally evaluated in the dual culture assay.

### Fungal identification: DNA extraction, PCR, and sequencing

Fresh mycelium (100 mg) from each isolate was homogenized in 2 mL tubes containing ceramic beads using a FastPrep instrument (MP Biomedicals, Irvine, USA) at 4.5 m/s for 20 s in two consecutive rounds. DNA was extracted using the NucleoSpin® Tissue Kit (Macherey–Nagel, Düren, Germany) according to the manufacturer’s instructions.

Species identity of the isolates was confirmed by Sanger sequencing of standard fungal barcode loci. The nuclear ribosomal internal transcribed spacer (ITS) region was amplified with primers ITS1/ITS4^61^. In addition, partial TEF1-α was amplified using EF1-728F/EF1-986R^62^, β-tubulin using Bt2a/Bt2b^63^, and RPB2 using fRPB2-5F/fRPB2-7cR^64^. Amplicons were sequenced and compared against reference databases (e.g., BLAST/GenBank and UNITE) to confirm species level assignments. *Cadophora luteo-olivacea* CZ_395 was confirmed using ITS, TEF1-α, β-tubulin and RPB2, and *T. atroviride* CZ_180 using ITS, TEF1-α and RPB2*.* PCR reactions (22 µL total volume) were prepared using the GoTaq® Flexi DNA Polymerase system (Promega, Madison, WI, USA) and included: 1 µL of each primer (10 µM), 0.2 µL of dNTP mix (10 mM each), 6 µL of 5 × Colorless GoTaq® Reaction Buffer, 2 µL of MgCl_2_ (25 mM), 2.6 µL of 5 × Green GoTaq® Loading Dye, 0.2 µL of GoTaq® polymerase, and nuclease free water to 22 µL. Thermal cycling consisted of an initial denaturation at 95 °C for 2 min, followed by 35 cycles of denaturation at 95 °C for 30 s, annealing at ITS 58 °C, TEF1-α 58 °C, BT 55 °C, RPB2 55 °C for 30 s, and extension at 72 °C for 60 s, with a final extension step at 72 °C for 5 min.

Amplification success was verified by electrophoresis on 1.3% agarose gels (Serva, Heidelberg, Germany). PCR products were purified using the NucleoSpin® Tissue Kit (Macherey–Nagel) and sequenced at Eurofins Genomics (Ebersberg, Germany).

Sanger sequencing results from fungal isolates were quality checked and visualized using Geneious Prime (v2024.0.5). Taxonomic identification was conducted using NCBI’s BLASTn algorithm (megablast), with an e-value threshold of 0.05 and a minimum identity of 98%^65^.

### Culture maintenance

Isolates were maintained on potato dextrose agar (PDA; HiMedia Laboratories, India) at 20 °C in darkness and subcultured every 10–14 days for *T. atroviride*, and every 20–30 days for *C. luteo-olivacea*.

#### Plant material and pathogenicity tests

Two year old containerized *Quercus robur* seedlings (nonundercut) were obtained from the Forest School Křtiny (Czech Republic) (Fig. 1) in May 2023. Prior to inoculation, seedlings were acclimatized for two weeks in pots with horticultural substrate under nethouse conditions.

Pathogenicity of *C. luteo-olivacea* CZ_395 was tested using a standardized stem inoculation procedure^66^. Briefly, 2 mm holes were made in the stem with a sterile cork borer and filled with mycelial plugs from a 20 day old culture grown on PDA. Wounds were sealed with sterile moistened cotton, aluminum foil, and Parafilm. Negative controls received sterile PDA plugs without mycelium. This assay represents an artificial wounded stem inoculation system designed to test whether *C. luteo-olivacea* CZ_395 can induce lesions and be re-isolated from inoculated *Q. robur* seedlings under controlled experimental conditions.

A total of 21 seedlings were inoculated (I1: n = 10; I2: n = 11), and 10 seedlings served as controls (K: n = 10). I1 and I2 denote two inoculated subsets processed separately under the same protocol; K denotes wounded controls receiving sterile PDA plugs. Plants were maintained for eight months post inoculation in an insect proof nethouse under ambient weather conditions. Seedlings were harvested in February 2024, washed, photographed, and cut longitudinally.

Lesion length was defined as the visible longitudinal extent of dark stem discoloration/necrotic tissue extending from the inoculation point after cutting the stem. Lesion length was measured in cm and used as the primary quantitative measure of symptom severity. Plant length was also recorded to account for differences in seedling size. Lesion severity was additionally expressed as the proportion of plant length affected (% of plant length) as a complementary normalized descriptor.

Lesion length was analysed using a linear model with inoculation treatment as the main explanatory variable and plant length as a covariate. The two inoculated subsets were combined for the primary treatment level comparison between *C. luteo-olivacea* inoculated seedlings and controls. Relative lesion severity (% plant length affected) was analysed as a complementary sensitivity analysis.

Re-isolation was performed from symptomatic stem tissue to support Koch’s postulates. Tissue segments were excised from the visible necrotic lesion extending from the inoculation point, preferably from the lesion margin. The segments were surface-sterilized (70% ethanol, 30 s; 2% sodium hypochloride, 2 min; three rinses in sterile water), plated on PDA, and incubated at 20 °C. Emerging colonies were subcultured and identified by multilocus sequencing (ITS, TEF1-α, RPB2).

#### Dual culture interaction assay

Dual culture assays were performed in 90 mm Petri dishes containing potato dextrose agar (PDA; HiMedia Laboratories LLC, Kennett Square, PA, USA) overlaid with sterile cellophane to enable mycelial harvesting. To account for differences in growth rates, *C. luteo-olivacea* CZ_395 was pregrown on PDA for 14 days before establishing the dual culture. Day 0 (0 dpi) was defined as the placement of a 5 mm agar plug of *T. atroviride* CZ_180 from the actively growing colony margin on the opposite side of the plate. Monoculture plates of each isolate were prepared as growth references. Because monoculture and dual culture plates differed in colony arrangement, monoculture comparisons were interpreted as assay specific references for visible colony development.

Colony development was monitored by repeated plate photography from 20 February to 8 April 2024. During the active interaction phase, plates were photographed approximately every 2–3 days, followed by a final late time point. For the dual culture assay, sampling times were expressed relative to the placement of the *T. atroviride* plug, with measurements taken from 2 to 31 dpi.

For image based growth assessment, plates were photographed with a ruler included in each image as a scale reference. Each photograph was calibrated in ImageJ using the ruler, and the visible mycelial area of each colony was measured in cm^2^. Plate coverage (%) was calculated as the measured mycelial area divided by the total Petri dish area and multiplied by 100. Thus, plate coverage represents an ImageJ-based area measurement normalized to plate size and was not inferred from radial growth.

Colony radius was measured separately and used to calculate the percent inhibition of radial growth (PIRG) of *C. luteo-olivacea* in dual culture relative to monoculture according to the formula:

$$PIRG\left(\%\right)= \frac{{R}_{c}-{R}_{t}}{{R}_{c}}\times 100$$

where R_c_ is the radial growth of *C. luteo-olivacea* in monoculture and R_t_ is the radial growth of *C. luteo-olivacea* in dual culture.

All assays were performed with five biological replicates per treatment. Growth data were analysed separately for each sampling time point using Welch’s two sample t-test to compare monoculture and dual culture. Because the same monoculture versus dual culture comparison was repeated across multiple sampling time points, *p* values were adjusted using the BH-FDR procedure across time points within each organism and measured variable.

#### Proteomic and metabolite sampling and co-extraction

**Fig. 7:**
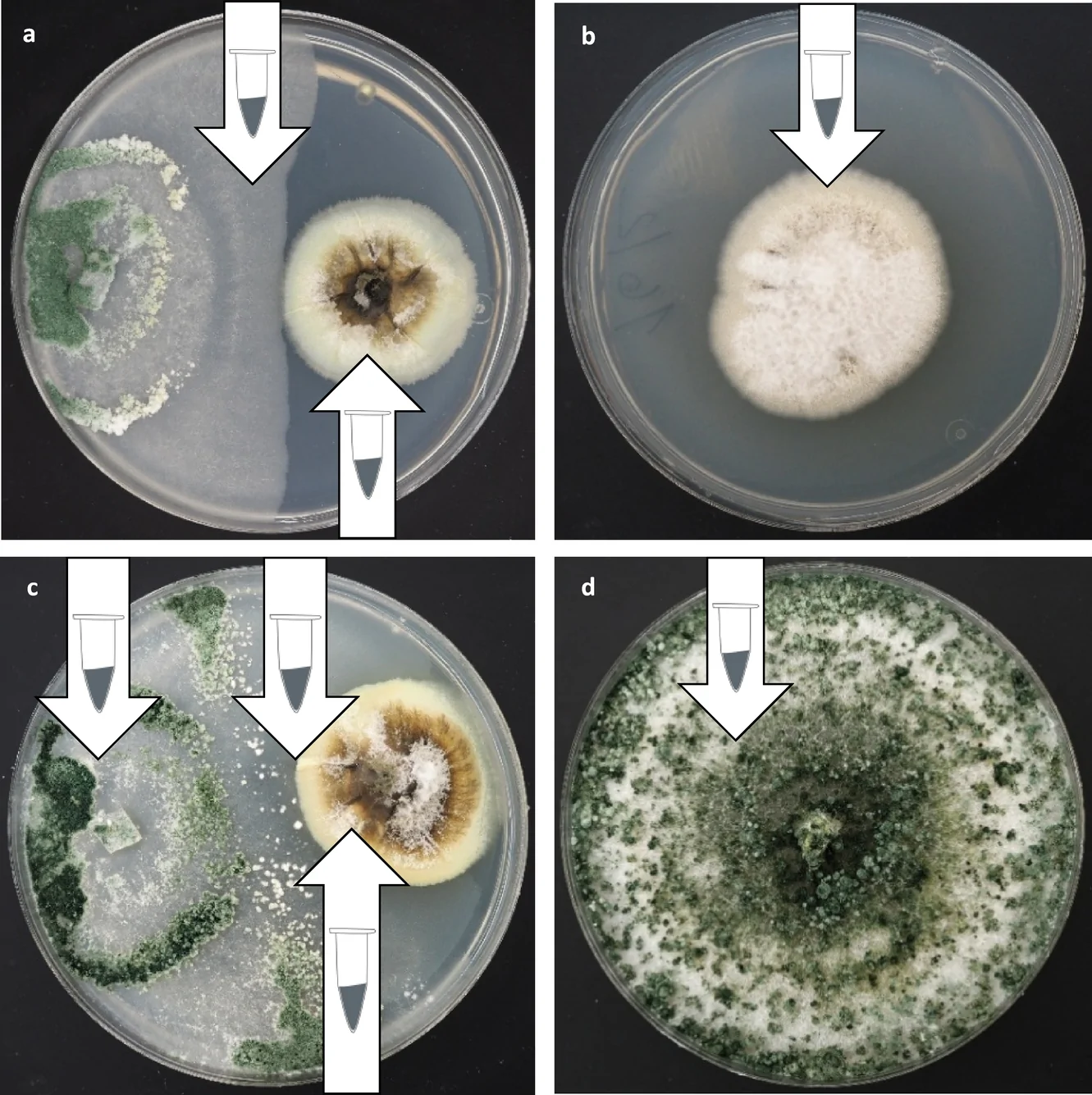
Mycelial sampling scheme in the interaction assay. Arrows indicate positions where mycelial plugs were excised. Sample input masses are provided in Supplementary Table S1. (**a**) interaction (INT; dual culture) at 4 days post inoculation (dpi): sampling across the whole colonies of Trichoderma and Cadophora. (**b**) Cadophora MOCK (monoculture): sampling at 4 and 8 dpi from the corresponding colony region. (**c**) INT at 8 dpi: sampling from the contact zone (interaction area) and a distant zone (away from the interaction). (**d**) Trichoderma MOCK (monoculture): sampling at 4 and 8 dpi from the corresponding colony region.

To investigate protein level responses during antagonism, mycelial tissue was sampled from dual cultures of *C. luteo-olivacea* (CZ_395) and *T. atroviride* (CZ_180) at 4 and 8 dpi after hyphal contact (n = 4, per treatment and timepoint). At each timepoint, mycelium was sampled from the confrontation zone and distant zone of dual cultures, together with corresponding monoculture reference samples grown on the same medium and cellophane overlay and incubated under the same environmental condition (Fig. 7). This design was used to distinguish protein abundance patterns associated with the interaction zone from those observed in non-contact mycelium and monoculture reference material.

Mycelia were lyophilized and homogenized in a RETSCH mill using grinding balls (5 min, 30 Hz). Homogenized material was extracted with 1 mL precooled (− 20 °C) MTBE/methanol (3:1, v/v) spiked with [^13^C]-L-valine, following the co-extraction protocol described in^67^ with minor modifications. Extracts were vortexed (RETSCH mill; 1 min, 30 Hz; precooled rack), sonicated for 15 min in an ice cooled bath, and centrifuged for 10 min at 10,000 g (4 °C). The supernatant was transferred to new tubes, while the remaining pellet was washed with 0.55 mL methanol, recentrifuged (10 min, 10,000 g, 4 °C), and the supernatants were combined. A 600 µL aliquot of the combined extract was transferred to a new tube, followed by addition of 150 µL MTBE and 450 µL H_2_O to induce phase separation. After vortexing (1 min, 30 Hz) and centrifugation (10 min, 10,000 g, 4 °C), the upper (nonpolar) and lower (polar) phases were collected for GC–MS analysis and dried in a speed vacuum concentrator. The remaining protein rich pellet was washed with 80% acetone (v/v) and stored at − 80 °C for proteomic processing.

#### Proteomics (LC–MS/MS)

Protein extraction from the protein rich pellet followed^68^. Briefly, pellets were resolubilized in 2% (w/v) SDS, 30% (w/v) sucrose, 5% (v/v) β-mercaptoethanol, 5 mM EDTA, and 100 mM Tris (pH 8.0), supplemented with 400 µL Tris saturated phenol. Proteins were precipitated from the phenolic phase using 100 mM ammonium acetate in methanol, washed with 80% acetone, and solubilized in 8 M urea, 10 mM DTT, and 100 mM ammonium bicarbonate. Proteins were alkylated and digested with trypsin. Each sample (5 µg peptides) was analyzed by nanoflow reverse phase LC–MS/MS using a 25 cm bioZen 2.6 µm Peptide XBC18 (Phenomenex) column coupled to a Dionex Ultimate 3000 RSLC nano UPLC and an Orbitrap Fusion Lumos Tribrid Mass Spectrometer equipped with a FAIMS Pro interface (Thermo Fisher Scientific)^69^, using alternating FAIMS compensation voltages of − 40, − 50, and − 75 V. The mass spectrometry proteomics data have been deposited to the ProteomeXchange Consortium via the PRIDE partner repository^70^ with the dataset identifier PXD073144.

Protein sequence databases (FASTA): For *Trichoderma atroviride*, the reference proteome was downloaded from UniProtKB (strain ATCC 20,476/IMI 206,040 reviewed and unreviewed entries; accessed 22 September 2023). For *Cadophora luteo-olivacea*, a predicted proteome derived from an annotated whole genome sequence (strain MEND-F-0454; Mendeleum collection) was used, as no publicly curated proteome was available at the time. Common contaminants were appended, and decoy sequences were included for FDR estimation.

MS/MS spectra were recalibrated and searched against the corresponding species specific protein database using Proteome Discoverer 2.5 with Sequest HT, MS Amanda 2.0^71^, and MSFragger^72^. Quantification was restricted to proteins identified by at least two unique peptides. In addition, de novo assisted interpretation was performed using PEAKS 6.0 and Unipept for downstream interpretation^73^.

#### Metabolomics (GC–MS)

Dried polar and nonpolar fractions were derivatized by methoximation (40 mg methoxyamine hydrochloride in 1 mL pyridine; 90 min at 30 °C, shaking), followed by silylation (60 µL N-methyl-N-trimethylsilyltrifluoroacetamide; 30 min at 37 °C, shaking) and analyzed by GC–MS. Derivatized metabolites were injected onto a TG-5SILMS GC column (30 m × 0.25 mm × 0.25 µm; Thermo Fisher) and separated using a 28 min temperature gradient (70–320 °C). Ionization was performed in EI mode (70 eV; emission current 50 µA; transfer line and ion source 250 °C). Data were processed in Compound Discoverer 3.3 (Thermo Fisher; peak detection 5 ppm; TIC threshold 25,000; S/N threshold 3) and searched against NIST2023, the GC-Orbitrap metabolomics library, and an in house library. Only metabolites meeting identification criteria (score > 80 and ΔRI < 1%) were retained. Quantitative differences were validated by manual peak inspection in Skyline 19.1^74^.

#### Functional annotation and classification

Protein functional annotation was performed using UniProtKB^75^. nterPro/Pfam signatures and Gene Ontology terms were retrieved with InterProScan v5^76^. Each protein was assigned to a single top level functional class using a fixed priority rule based on UniProt keywords, GO, and InterPro/Pfam; composition plots report the per condition percentage of total abundance per class. Carbohydrate active enzymes were annotated at the family level using CAZy-supported mappings from InterPro/Pfam^77^. Over representation analyses were performed for GO (MF, CC) and InterPro on upregulated sets in each contrast, using the quantified proteome as background and Benjamini Hochberg correction^78^.

### Statistical analysis

Analyses were performed in Python 3.9 using (pandas, NumPy, SciPy/statsmodels, matplotlib)^79,80^. Protein relative abundance values were log_2_ transformed before statistical testing and visualization. For each protein and contrast, log_2_ fold changes were calculated as the difference between group means on the log_2_ scale. Differentially abundant proteins (DAPs) were identified using two sided Welch’s t-tests applied to log_2_ transformed relative abundances, with BH-FDR correction applied across proteins within each contrast. Proteins were classified as DAPs when they met both criteria: FDR < 0.05 and |log_2_FC|≥ 0.58, corresponding to an absolute fold change of at least 1.5. The fold change threshold was used as an effect size filter to retain statistically supported changes of at least moderate magnitude, rather than as an independent biological cutoff. The same DAP criteria were applied consistently across proteomic contrasts and time points. Protein level results were interpreted as candidate abundance changes because of the limited number of biological replicates.

For GO composition summaries, proteins assigned to multiple cellular component terms were fractionally assigned across terms. For each condition, the summed abundance assigned to each GO term was expressed as a percentage of the total GO protein abundance. Δ share values were calculated as percentage-point differences relative to the corresponding mock condition.

For metabolomics, peak areas were first normalized to sample dry mass and subsequently median normalized across samples to reduce global intensity differences, followed by log_2_ transformation. Multivariate structure was evaluated by PCA. Group dispersion in ordination space was assessed by PERMDISP (distance to group centroid). Differential metabolites were tested per contrast using two sided Welch’s t-tests for unpaired contrasts and paired t-tests for replicate paired contrasts, followed by BH-FDR correction across metabolites within each contrast. Metabolites with FDR < 0.05 were considered significant.

## Supplementary Information

Below is the link to the electronic supplementary material.

Supplementary Material 1

Supplementary Material 2

## Data Availability

The mass spectrometry proteomics data have been deposited to the ProteomeXchange Consortium (via PRIDE) under the dataset identifier PXD073144. The nucleotide sequences generated for isolate verification (ITS, TEF1-α, RPB2) are available in GenBank under the following accession numbers: *Cadophora luteo-olivacea* CZ_395 (PX843264–PX843267; PX898111–PX898114; PX898115–PX898120; PX842612) and *Trichoderma atroviride* CZ_180 (PX927109; PX842629; PX898121–PX898122). Processed proteomics and metabolomics result tables supporting the findings are provided in the Supplementary Tables.

## Abbreviations

ABC: ATP-binding cassette (transporters)
ABCG: ATP-binding cassette, subfamily G
ANOVA: Analysis of variance
ATCC: American Type Culture Collection
BH/BH-FDR: Benjamini Hochberg/Benjamini Hochberg false discovery rate control
BLASTn: Basic Local Alignment Search Tool (nucleotide)
BT: β-tubulin
CAZy: Carbohydrate-Active enZymes (database)
CAZymes: Carbohydrate-active enzymes
CBM: Carbohydrate-binding module
CBM50/LysM: LysM (lysin motif) carbohydrate-binding module
CBS: Cystathionine β-synthase
CC (GO:CC): Cellular Component (Gene Ontology)
cvm (CVM): Contact versus mock (contrast)
cvd (CVD): Contact versus distant (contrast; in text replicate-paired at 8 dpi)
DAPs: Differentially abundant proteins
DNA: Deoxyribonucleic acid
dNTPs: Deoxynucleoside triphosphates
DTT: Dithiothreitol
dvm (DVM): Distant versus mock (contrast)
dpi: Days post inoculation
EDTA: Ethylenediaminetetraacetic acid
EF1/TEF1-α: Translation elongation factor 1-alpha
EI: Electron ionization
EPL2: Ceratoplatanin/EPL protein (eliciting plant response-like; *Trichoderma*)
FAIMS: High-field asymmetric waveform ion mobility spectrometry
FASTA: FASTA sequence format
FDR: False discovery rate
FR-type: Ferric(-chelate) reductase type
GC–MS: Gas chromatography mass spectrometry
GH: Glycoside hydrolase (CAZy family; e.g., GH18, GH17, GH3)
GO: Gene ontology
GPCR: G-protein-coupled receptor
HSD: Honestly significant difference (Tukey’s HSD)
HTAS: High throughput amplicon sequencing
IMI: Culture collection strain identifier (IMI)
InterPro/IPR: InterPro/InterPro accession (e.g., IPR000182)
InterProScan: InterProScan software
INT: Interaction (dual culture) condition
ITS: Internal transcribed spacer
LC–MS/MS: Liquid chromatography tandem mass spectrometry
log_2_FC: Log2 fold change
LPMO: Lytic polysaccharide monooxygenase
MAPK: Mitogen activated protein kinase
MF (GO:MF): Molecular Function (Gene Ontology)
MFS: Major facilitator superfamily (transporters)
MOCK: Mock control (monoculture baseline)
MS/MS: Tandem mass spectrometry
MTAP: Methylthioadenosine phosphorylase
MTBE: Methyl tert-butyl ether
NCBI: National Center for Biotechnology Information
NIST: National Institute of Standards and Technology
PCA: Principal component analysis
PCoA: Principal coordinates analysis
PDA: Potato dextrose agar
PDR: Pleiotropic drug resistance (ABCG-type efflux transporters)
PERMDISP: Permutational analysis of multivariate dispersions
Pfam: Protein families database
PIRG: Percent inhibition of radial growth
PKS: Polyketide synthase
PRIDE: PRoteomics IDEntifications Database
ProteomeXchange: ProteomeXchange Consortium (repository framework)
PXD: ProteomeXchange dataset identifier prefix
QC: Quality control
RI/ΔRI: Retention index/delta retention index
RNA: Ribonucleic acid
RPB2: RNA polymerase II second largest subunit
ROS: Reactive oxygen species
S/N: Signal to noise ratio
SDS: Sodium dodecyl sulfate
TDPs: Trunk disease pathogens
TDs: Trunk diseases
TEF1-α: Translation elongation factor 1-alpha
TIC: Total ion current
Tris: Tris(hydroxymethyl)aminomethane (buffer)
UPLC: Ultra-performance liquid chromatography
UniProtKB: UniProt Knowledgebase
δ (Cliff’s δ): Cliff’s delta (effect size)

## Code availability

The analysis scripts, processed input tables, and the script used to generate Fig. 6 are available in the GitHub repository https://github.com/Frey-Lu/cadophora_trichoderma_multiomics and archived in Zenodo under the version-specific https://doi.org/10.5281/zenodo.20721711.

## References

[1] Eaton, E., Caudullo, G., Oliveira, S. & de Rigo, D. *Quercus robur* and *Quercus petraea* in Europe: Distribution, habitat, usage and threats. In *European Atlas of Forest Tree Species* (eds. San-Miguel-Ayanz, J., de Rigo, D., Caudullo, G., Houston Durrant, T. & Mauri, A.) e01c6df (Publ. Off. EU, Luxembourg, 2016).

[2] Krutovsky, K. V., Popova, A. A., Yakovlev, I. A., Yanbaev, Y. A. & Matveev, S. M. Response of Pedunculate Oak (*Quercus robur* L.) to Adverse environmental conditions in genetic and dendrochronological studies. *Plants* **14**, 109 (2025). 39795368 10.3390/plants14010109 PMC11723010

[3] Mitchell, R. J. et al. Collapsing foundations: The ecology of the British oak, implications of its decline and mitigation options. *Biol. Cons.* **233**, 316–327 (2019).

[4] Mölder, A. et al. Success factors for high-quality oak forest (*Quercus robur*, *Q. petraea*) regeneration. *For. Ecosyst.* **6**, 49 (2019).

[5] Forest Europe. *State of Europe’s Forests 2020: Status and Trends in Sustainable Forest Management in Europe*. (Bratislava, 2020).

[6] Freer-Smith, P. et al. *Plantation Forests in Europe: Challenges and Opportunities*. https://www.efi.int/publications-bank/plantation-forests-europe-challenges-and-opportunities (2019). .

[7] *Spruce Monocultures in Central Europe: Problems and Prospects*. (European Forest Institute, Joensuu, 2000).

[8] Löf, M., Bergquist, J., Brunet, J., Karlsson, M. & Welander, N. T. Conversion of Norway spruce stands to broadleaved woodland-regeneration systems, fencing and performance of planted seedlings. *Ecol. Bull. ***53**, 165–174 (2010).

[9] Singh, V. V. et al. Understanding bark beetle outbreaks: exploring the impact of changing temperature regimes, droughts, forest structure, and prospects for future forest pest management. *Rev. Environ. Sci. Biotechnol.* **23**, 257–290 (2024).

[10] Spiecker, H. Silvicultural management in maintaining biodiversity and resistance of forests in Europe—temperate zone. *J. Environ. Manag.* **67**, 55–65 (2003). 10.1016/s0301-4797(02)00188-3 12659804

[11] Mora-Sala, B. et al. Survey, identification, and characterization of cylindrocarpon-like asexual morphs in Spanish forest nurseries. *Plant Dis.* **102**, 2083–2100 (2018). 30189159 10.1094/PDIS-01-18-0171-RE

[12] Wingfield, M. J., Brockerhoff, E. G., Wingfield, B. D. & Slippers, B. Planted forest health: The need for a global strategy. *Science* **349**, 832–836 (2015). 26293956 10.1126/science.aac6674

[13] Balla, A. et al. The Threat of pests and pathogens and the potential for biological control in forest ecosystems. *Forests* **12**, 1579 (2021).

[14] Calvo-Garrido, C. et al. Description of the relationship between trunk disease expression and meteorological conditions, irrigation and physiological response in Chardonnay grapevines. *OENO One* **55**, 97–113 (2021).

[15] Gramaje, D. et al. Fungal trunk diseases: A problem beyond grapevines?. *Plant. Pathol.* **65**, 355–356 (2016).

[16] Eichmeier, A. et al. Fungal trunk pathogens associated with *Juglans regia* in the Czech Republic. *Plant Dis.* **104**, 761–771 (2020). 31944904 10.1094/PDIS-06-19-1308-RE

[17] Mahamedi, A. E. et al. Diversity, distribution and host association of *Botryosphaeriaceae* species causing oak decline across different forest ecosystems in Algeria. *Eur. J. Plant Pathol.* **158**, 745–765 (2020).

[18] Gu, Y. et al. Small changes in rhizosphere microbiome composition predict disease outcomes earlier than pathogen density variations. *ISME J* **16**, 2448–2456 (2022). 35869387 10.1038/s41396-022-01290-z PMC9478146

[19] Mannaa, M. & Seo, Y.-S. Plants under the attack of allies: Moving towards the plant pathobiome paradigm. *Plants* **10**, 125 (2021). 33435275 10.3390/plants10010125 PMC7827841

[20] Olanrewaju, O. S., Glick, B. R. & Babalola, O. O. Beyond correlation: Understanding the causal link between microbiome and plant health. *Heliyon* **10**, e40517 (2024). 39669148 10.1016/j.heliyon.2024.e40517 PMC11636107

[21] Schlatter, D., Kinkel, L., Thomashow, L., Weller, D. & Paulitz, T. Disease suppressive soils: New insights from the soil microbiome. *Phytopathology* **107**, 1284–1297 (2017). 28650266 10.1094/PHYTO-03-17-0111-RVW

[22] Stewart, J. E., Kim, M.-S., Lalande, B. & Klopfenstein, N. B. Pathobiome and microbial communities associated with forest tree root diseases. In: Asiegbu, Fred O.; Kovalchuk, Andriy, eds. *Forest Microbiology* - Tree Microbiome: Phyllosphere, Endosphere, and Rhizosphere, Volume 1. London, UK: Academic Press, p. 277–292 (Elsevier, 2021). 10.1016/B978-0-12-822542-4.00004-8

[23] Di Francesco, A. et al. Pseudomonas synxantha volatile organic compounds: Efficacy against *Cadophora luteo-olivacea* and *Botrytis cinerea* of kiwifruit. *Front. Plant Sci.* **15**, 1398014 (2024). 38779078 10.3389/fpls.2024.1398014 PMC11109433

[24] Di Francesco, A., Di Foggia, M., Fasano, A. & Baraldi, E. Heat treatment effect on *Cadophora luteo-olivacea* of kiwifruit. *Plant. Pathol.* **71**, 644–653 (2022).

[25] Maldonado-González, M. M. et al. Quantification of *Cadophora luteo-olivacea* from grapevine nursery stock and vineyard soil using droplet digital PCR. *Plant Dis* **104**, 2269–2274 (2020). 32568630 10.1094/PDIS-09-19-2035-RE

[26] Tomada, S., Staffler, E., Dionis, G., Baric, S. & Di Francesco, A. Cadophora luteo-olivacea on apple and kiwifruit: Characterization of selected strains and evaluation of fungicides for their control. *Eur. J. Plant Pathol.* **169**, 99–111 (2024).

[27] Travadon, R., Lawrence, D. P., Moyer, M. M., Fujiyoshi, P. T. & Baumgartner, K. Fungal species associated with grapevine trunk diseases in Washington wine grapes and California table grapes, with novelties in the genera Cadophora, Cytospora, and Sporocadus. *Front. Fungal Biol.* **3**, 1018140 (2022). 37746176 10.3389/ffunb.2022.1018140 PMC10512239

[28] Travadon, R. et al. Cadophora species associated with wood-decay of grapevine in North America. *Fungal Biol.* **119**, 53–66 (2015). 25601149 10.1016/j.funbio.2014.11.002

[29] Vicente, J., Alonso, A., Navascués, E., Marquina, D. & Santos, A. Specific and sensitive PCR detection of *Cadophora luteo-olivacea* associated with grapevine trunk diseases. *Crop Prot.* **132**, 105140 (2020).

[30] Manoharachary, C., Singh, H. B. & Varma, A. (eds) Trichoderma: Agricultural Applications and Beyond. Soil Biology Vol. 61 (Springer, Cham, 2020). 10.1007/978-3-030-54758-5.

[31] Schuster, A. & Schmoll, M. Biology and biotechnology of Trichoderma. *Appl. Microbiol. Biotechnol.* **87**, 787–799 (2010). 20461510 10.1007/s00253-010-2632-1 PMC2886115

[32] Vinale, F. et al. *Trichoderma*–plant–pathogen interactions. *Soil Biol. Biochem.* **40**, 1–10 (2008).

[33] Nogueira-Lopez, G. et al. The apoplastic secretome of *Trichoderma virens* during interaction with maize roots shows an inhibition of plant defence and scavenging oxidative stress secreted proteins. *Front. Plant Sci.* **9**, 409 (2018). 29675028 10.3389/fpls.2018.00409 PMC5896443

[34] Ramada, M. H. S., Steindorff, A. S., Bloch, C. & Ulhoa, C. J. Secretome analysis of the mycoparasitic fungus *Trichoderma harzianum* ALL 42 cultivated in different media supplemented with *Fusarium solani* cell wall or glucose. *Proteomics* **16**, 477–490 (2016). 26631988 10.1002/pmic.201400546

[35] Suarez, M. B. et al. Proteomic analysis of secreted proteins from *Trichoderma harzianum*. *Fungal Genet. Biol.* 10.1016/j.fgb.2005.08.002 (2005). 16226906

[36] Todd, J. N. A. et al. Proteomics-based prediction of candidate effectors in the interaction secretome of *Trichoderma harzianum* and *Pseudocercospora fijiensis*. *Microbiol. Res.* **16**, 175 (2025).

[37] Mayo-Prieto, S. et al. Effect of *Trichoderma velutinum* and *Rhizoctonia solani* on the metabolome of bean plants (*Phaseolus vulgaris* L.). *Int. J. Mol. Sci.* **20**, 549 (2019). 30696057 10.3390/ijms20030549 PMC6387467

[38] Schweiger, R. et al. Insights into metabolic changes caused by the *Trichoderma virens*—maize root interaction. *MPMI* **34**, 524–537 (2021). 33166203 10.1094/MPMI-04-20-0081-R

[39] Zeilinger, S., Gruber, S., Bansal, R. & Mukherjee, P. K. Secondary metabolism in Trichoderma—chemistry meets genomics. *Fungal Biol. Rev.* **30**, 74–90 (2016).

[40] Frejlichová, L., Maldonado-González, M. M., Škarpa, P., Tomšovský, M. & Eichmeier, A. *Beyond detection: Unveiling microbial dynamics in oak seedlings using isolation and high-throughput amplicon sequencing*. Preprint at 10.21203/rs.3.rs-7760938/v1 (2025). PMC13628951 42421159

[41] Gramaje, D., Mostert, L. & Armengol, J. Characterization of *Cadophora luteo-olivacea* and *C. melinii* isolates obtained from grapevines and environmental samples from grapevine nurseries in Spain. *Phytopathol. Mediterr.* 10.14601/Phytopathol_Mediterr-8723 (2011).

[42] Kubicek, C. P. et al. Comparative genome sequence analysis underscores mycoparasitism as the ancestral life style of Trichoderma. *Genome Biol.* **12**, R40 (2011). 21501500 10.1186/gb-2011-12-4-r40 PMC3218866

[43] Mukherjee, P. K., Mendoza-Mendoza, A., Zeilinger, S. & Horwitz, B. A. Mycoparasitism as a mechanism of Trichoderma-mediated suppression of plant diseases. *Fungal Biol. Rev.* **39**, 15–33 (2022).

[44] Faust, K. & Raes, J. Microbial interactions: From networks to models. *Nat. Rev. Microbiol.* **10**, 538–550 (2012). 22796884 10.1038/nrmicro2832

[45] Röttjers, L. & Faust, K. From hairballs to hypotheses–biological insights from microbial networks. *FEMS Microbiol. Rev.* **42**, 761–780 (2018). 30085090 10.1093/femsre/fuy030 PMC6199531

[46] Weiss, S. et al. Correlation detection strategies in microbial data sets vary widely in sensitivity and precision. *ISME J.* **10**, 1669–1681 (2016). 26905627 10.1038/ismej.2015.235 PMC4918442

[47] Tanney, J. B., Kemler, M., Vivas, M., Wingfield, M. J. & Slippers, B. Silent invaders: The hidden threat of asymptomatic phytobiomes to forest biosecurity. *New Phytol.* **247**, 533–545 (2025). 40400211 10.1111/nph.70209 PMC12177268

[48] Atanasova, L. et al. Comparative transcriptomics reveals different strategies of Trichodermamycoparasitism. *BMC Genomics* **14**, 121 (2013). 23432824 10.1186/1471-2164-14-121 PMC3599271

[49] Kappel, L. et al. A comparative cell wall analysis of *Trichoderma* spp. confirms a conserved polysaccharide scaffold and suggests an important role for chitosan in mycoparasitism. *Microbiol. Spectr.* **12**, e03495-23 (2024). 38916333 10.1128/spectrum.03495-23 PMC11302013

[50] Lesage, G. & Bussey, H. Cell wall assembly in *Saccharomyces cerevisiae*. *Microbiol. Mol. Biol. Rev.* **70**, 317–343 (2006). 16760306 10.1128/MMBR.00038-05 PMC1489534

[51] Levin, D. E. Cell wall integrity signaling in *Saccharomyces cerevisiae*. *Microbiol. Mol. Biol. Rev.* **69**, 262–291 (2005). 15944456 10.1128/MMBR.69.2.262-291.2005 PMC1197416

[52] Ortiz-Ramírez, J. A., Cuéllar-Cruz, M. & López-Romero, E. Cell compensatory responses of fungi to damage of the cell wall induced by Calcofluor White and Congo Red with emphasis on *Sporothrix schenckii* and *Sporothrix globose*. A review. *Front. Cell. Infect. Microbiol.* **12**, 976924 (2022). 36211971 10.3389/fcimb.2022.976924 PMC9539796

[53] Su, X. et al. Calcofluor white hypersensitive proteins contribute to stress tolerance and pathogenicity in entomopathogenic fungus *Metarhizium acridum*. *Pest Manag. Sci.* **77**, 1915–1924 (2021). 33300230 10.1002/ps.6218

[54] Hu, Z. et al. Recent advances in ergosterol biosynthesis and regulation mechanisms in *Saccharomyces cerevisiae*. *Indian J. Microbiol.* **57**, 270–277 (2017). 28904410 10.1007/s12088-017-0657-1 PMC5574775

[55] Kodedová, M. & Sychrová, H. Changes in the Sterol composition of the plasma membrane affect membrane potential, salt tolerance and the activity of multidrug resistance pumps in *Saccharomyces cerevisiae*. *PLoS ONE* **10**, e0139306 (2015). 26418026 10.1371/journal.pone.0139306 PMC4587746

[56] Khan, R. A. A., Najeeb, S., Hussain, S., Xie, B. & Li, Y. Bioactive secondary metabolites from *Trichoderma* spp. against phytopathogenic fungi. *Microorganisms* **8**, 817 (2020). 32182971 10.3390/microorganisms8030401 PMC7143365

[57] Khan, R. A. A. et al. Bioactive secondary metabolites from *Trichoderma* spp. against phytopathogenic bacteria and root-knot nematode. *Microorganisms* **8**, 401 (2020). 32182971 10.3390/microorganisms8030401 PMC7143365

[58] Patti, G. J., Yanes, O. & Siuzdak, G. Metabolomics: the apogee of the omics trilogy. *Nat. Rev. Mol. Cell Biol.* **13**, 263–269 (2012). 22436749 10.1038/nrm3314 PMC3682684

[59] Brodhun, F. & Feussner, I. Oxylipins in fungi. *FEBS J.* **278**, 1047–1063 (2011). 21281447 10.1111/j.1742-4658.2011.08027.x

[60] Niu, M. et al. Fungal oxylipins direct programmed developmental switches in filamentous fungi. *Nat. Commun.* **11**, 5158 (2020). 33056992 10.1038/s41467-020-18999-0 PMC7557911

[61] White, T., Burns, T., Lee, S. & Taylor, J. *Amplification and Direct Sequencing of Fungal Ribosomal RNA Genes for Phylogenetics* (Academic Press, 1990).

[62] Carbone, I. & Kohn, L. M. A method for designing primer sets for speciation studies in filamentous ascomycetes. *Mycologia* **91**, 553–556 (1999).

[63] Glass, N. L. & Donaldson, G. C. Development of primer sets designed for use with the PCR to amplify conserved genes from filamentous ascomycetes. *Appl. Environ. Microbiol.* **61**, 1323–1330 (1995). 7747954 10.1128/aem.61.4.1323-1330.1995 PMC167388

[64] Liu, Y. J., Whelen, S. & Hall, B. D. Phylogenetic relationships among ascomycetes: Evidence from an RNA polymerse II subunit. *Mol. Biol. Evol.* **16**, 1799–1808 (1999). 10605121 10.1093/oxfordjournals.molbev.a026092

[65] Stover, N. A. & Cavalcanti, A. R. O. Using NCBI BLAST. In *Current Protocols Essential Laboratory Techniques* 11.1.1–11.1.34, Vol. 14 (2017).

[66] Luque, J., Parlade, J. & Pera, J. Pathogenicity of fungi isolated from *Quercus suber* in Catalonia (NE Spain). *Forest Pathol* **30**, 247–263 (2000).

[67] Berková, V. et al. Salicylic acid treatment and its effect on seed yield and seed molecular composition of *Pisum sativum* under abiotic stress. *IJMS* **24**, 5454 (2023). 36982529 10.3390/ijms24065454 PMC10049190

[68] Berková, V. et al. The fungus *Acremonium alternatum* enhances salt stress tolerance by regulating host redox homeostasis and phytohormone signaling. *Physiol. Plant.* **176**, e14328 (2024). 38695265 10.1111/ppl.14328

[69] Kopecká, R. et al. HSP70 as a mediator of host-pathogen interaction in *Arabidopsis thaliana* during *Plasmodiophora brassicae* infection. *Physiol. Plant.* **177**, e70309 (2025). 40464190 10.1111/ppl.70309 PMC12135032

[70] Perez-Riverol, Y. et al. The PRIDE database resources in 2022: A hub for mass spectrometry-based proteomics evidences. *Nucleic Acids Res.* **50**, D543–D552 (2022). 34723319 10.1093/nar/gkab1038 PMC8728295

[71] Dorfer, V., Strobl, M., Winkler, S. & Mechtler, K. MS Amanda 2.0: Advancements in the standalone implementation. *Rapid Commun. Mass Spectrom.* **35**, e9088 (2021). 33759252 10.1002/rcm.9088 PMC8244010

[72] Kong, A. T., Leprevost, F. V., Avtonomov, D. M., Mellacheruvu, D. & Nesvizhskii, A. I. MSFragger: Ultrafast and comprehensive peptide identification in mass spectrometry-based proteomics. *Nat. Methods* **14**, 513–520 (2017). 28394336 10.1038/nmeth.4256 PMC5409104

[73] Chatterjee, S. et al. Protein paucimannosylation is an enriched N-glycosylation signature of human cancers. *Proteomics* **19**, 1900010 (2019). 10.1002/pmic.201900010 31419058

[74] Pino, L. K. et al. The Skyline ecosystem: Informatics for quantitative mass spectrometry proteomics. *Mass Spectrom. Rev.* **39**, 229–244 (2020). 28691345 10.1002/mas.21540 PMC5799042

[75] UniProt Consortium. UniProt: The Universal Protein Knowledgebase in 2023. *Nucleic Acids Res.* **51**, D523–D531 (2023). 10.1093/nar/gkac1052 PMC9825514 36408920

[76] Jones, P. et al. InterProScan 5: Genome-scale protein function classification. *Bioinformatics* **30**, 1236–1240 (2014). 24451626 10.1093/bioinformatics/btu031 PMC3998142

[77] Lombard, V., Golaconda Ramulu, H., Drula, E., Coutinho, P. M. & Henrissat, B. The carbohydrate-active enzymes database (CAZy) in 2013. *Nucleic Acids Res.* **42**, D490-495 (2014). 24270786 10.1093/nar/gkt1178 PMC3965031

[78] Benjamini, Y. & Hochberg, Y. Controlling the false discovery rate: A practical and powerful approach to multiple testing. *J. R. Stat. Soc. Ser. B Stat. Methodol.* **57**, 289–300 (1995).

[79] McKinney, W. *Data Structures for Statistical Computing in Python*. 56–61 (Austin, Texas, 2010). 10.25080/Majora-92bf1922-00a.

[80] Virtanen, P. et al. SciPy 1.0: Fundamental algorithms for scientific computing in Python. *Nat. Methods* **17**, 261–272 (2020). 32015543 10.1038/s41592-019-0686-2 PMC7056644

