# Supplementary material for: Pathogenicity of Cadophora luteo-olivacea on Quercus robur and multi-omics characterization of antagonism by Trichoderma atroviride
Source: Sci Rep. 2026 Jun 22;16:28564. doi: 10.1038/s41598-026-59030-8 (PMC13572560; doi:10.1038/s41598-026-59030-8)
Supplement: Supplementary file 1 [file 41598_2026_59030_MOESM1_ESM.pdf]

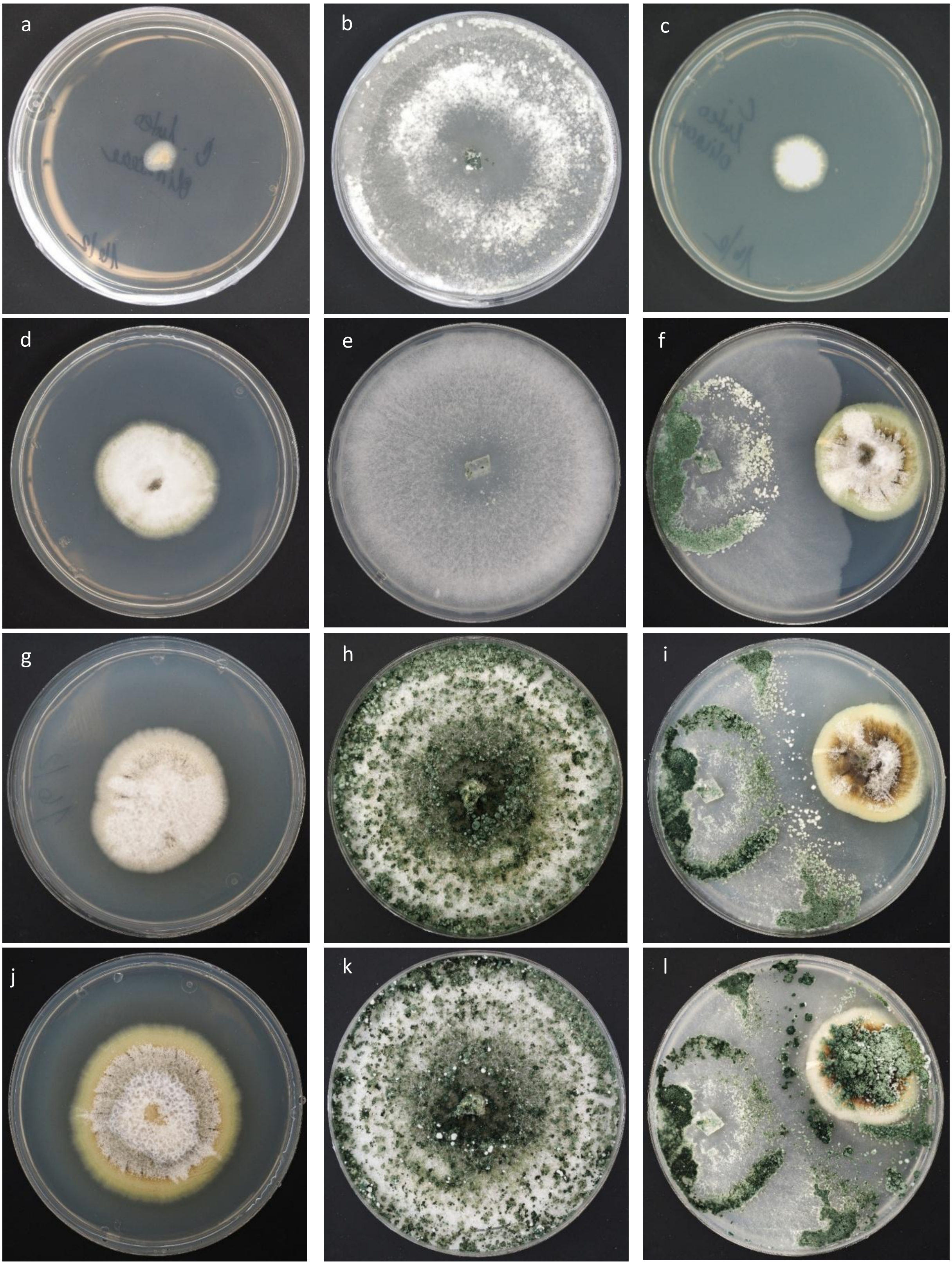

Figure S1: Colony development and mycelium sampling timeline for *Cadophora luteo-olivacea* and *Trichoderma atroviride* in monoculture and dual culture Table S4. (a) *C. luteo-olivacea*, 4 d. (b) *T. atroviride*, 4 d. (c) *C. luteo-olivacea*, 8 d. (d) Mycelium sampling: *C. luteo-olivacea*, 18 d. (e) Mycelium sampling: *T. atroviride*, 4 d. (f) Mycelium sampling (INT, 4 d): *T. atroviride* 4 d confronting *C. luteo-olivacea* 18 d. (g) Mycelium sampling: *C. luteo-olivacea*, 22 d. (h) Mycelium sampling: *T. atroviride*, 8 d. (i) Mycelium sampling (INT, 8 d): *T. atroviride* 8 d confronting *C. luteo-olivacea* 22 d. (j) *C. luteo-olivacea*, 29 d. (k) *T. atroviride*, 15 d. (l) Dual culture: *T. atroviride* 15 d confronting *C. luteo-olivacea* 29 d.

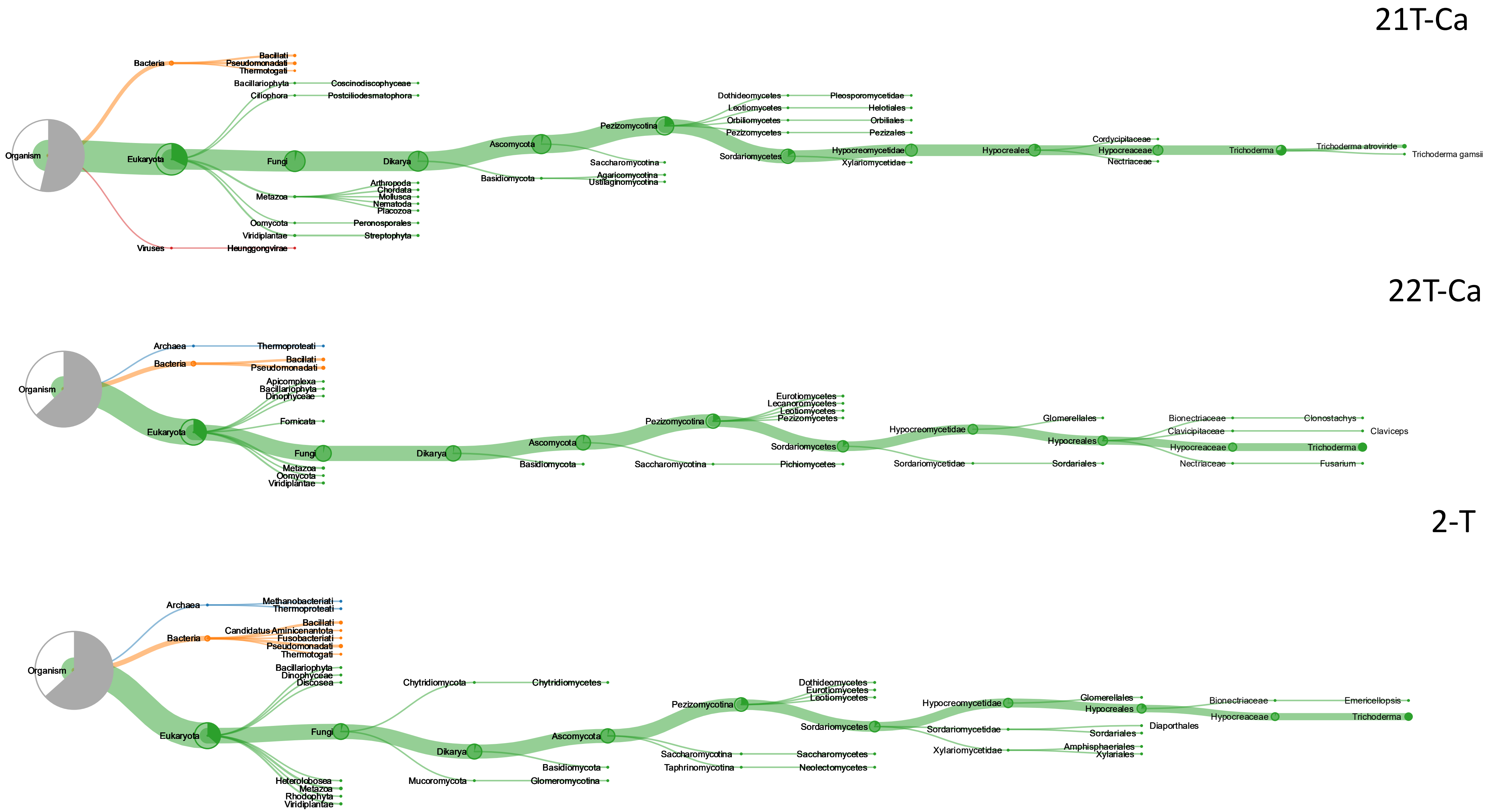

Figure S2: Metaproteomic taxonomic assignment confirms ***Trichoderma*** dominance in the proteomics samples. All identified peptides/proteins were taxonomically annotated and visualized as lineage flows, showing that the vast majority map to *Trichoderma* (Eukaryota → Fungi → Ascomycota → Hypocreales → *Trichoderma*), supporting correct species-level attribution of the interaction proteome.

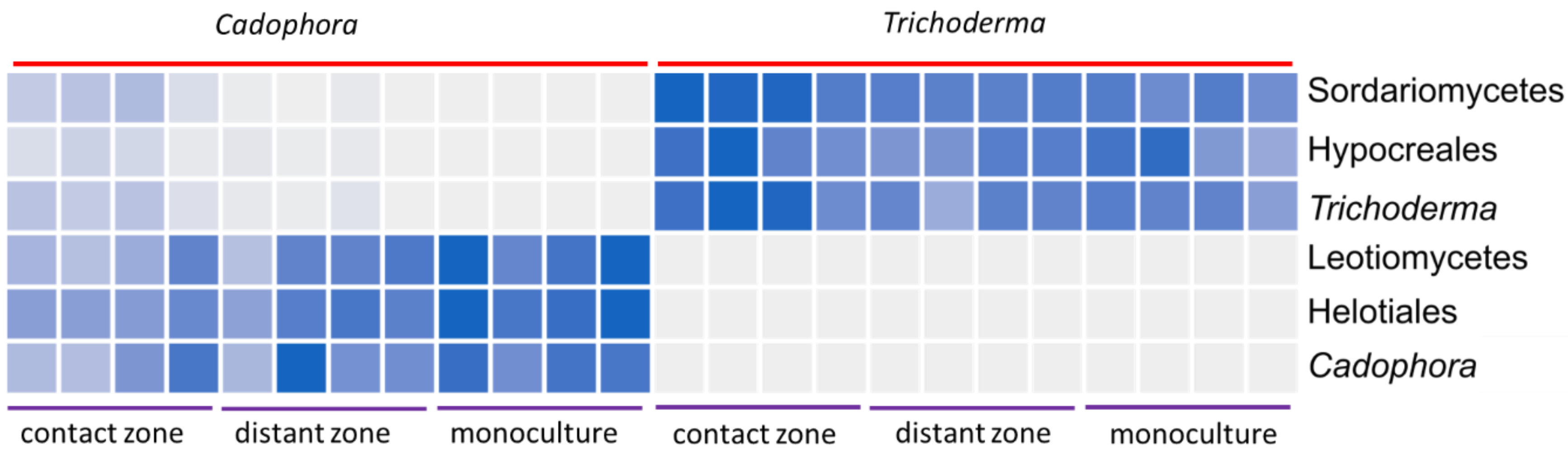

Figure S3: Taxonomic QC of de novo peptides (Unipept) De novo peptides (PEAKS 6.0) were assigned at genus level using Unipept. Monocultures showed genus consistent profiles, whereas Cadophora contact zone samples contained a detectable Trichoderma peptide contribution, indicating mixed species material in the confrontation area. Low confidence/rare off target assignments were omitted.

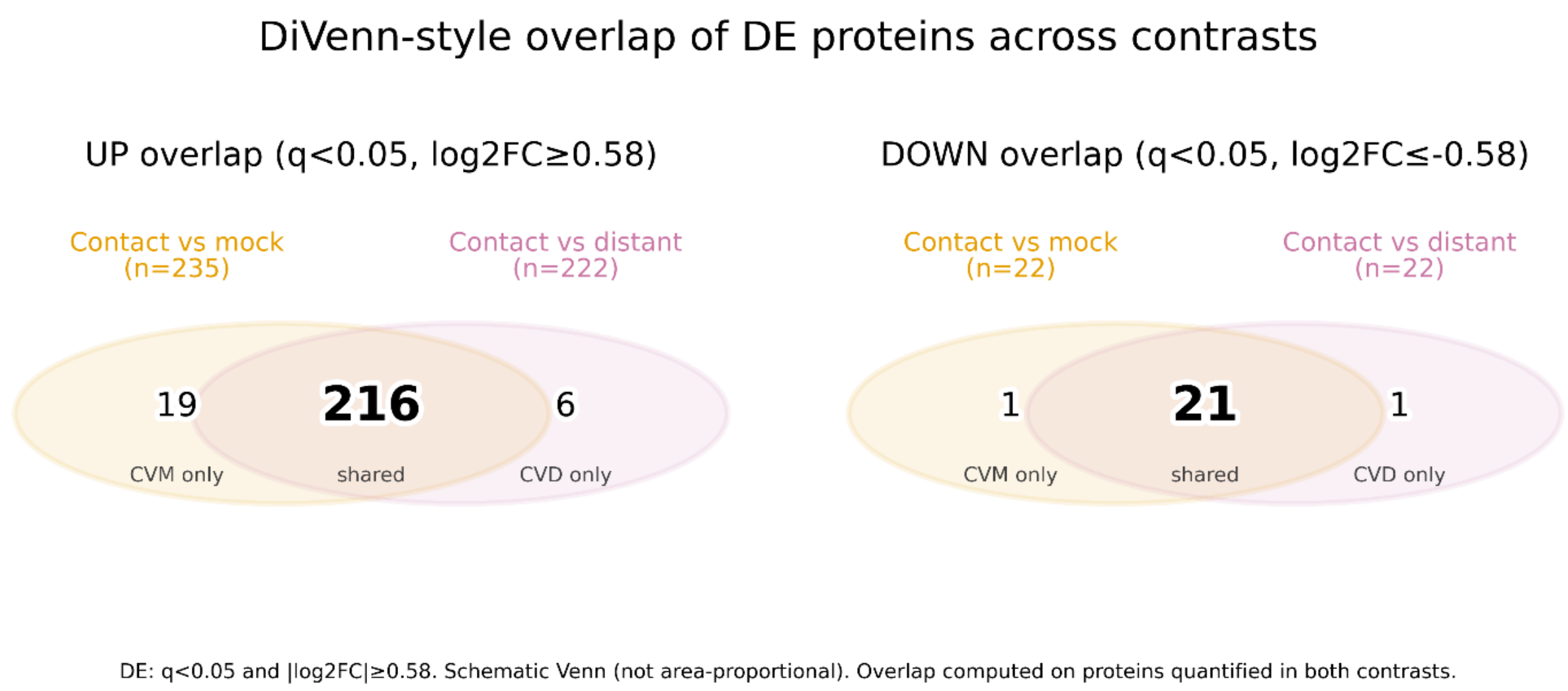

Figure S4: DiVenn style overlap of differentially abundant proteins using the same thresholds suplementary material (Table S8). Contact vs mock: 235 up / 22 down; contact vs distant: 222 up / 22 down. The overlap indicates that most DE proteins are shared between contrasts (216 up; 21 down), with only small contrast-specific subsets (UP: 19 cvm-only, 6 cvd-only; DOWN: 1 cvm-only, 1 cvd-only).

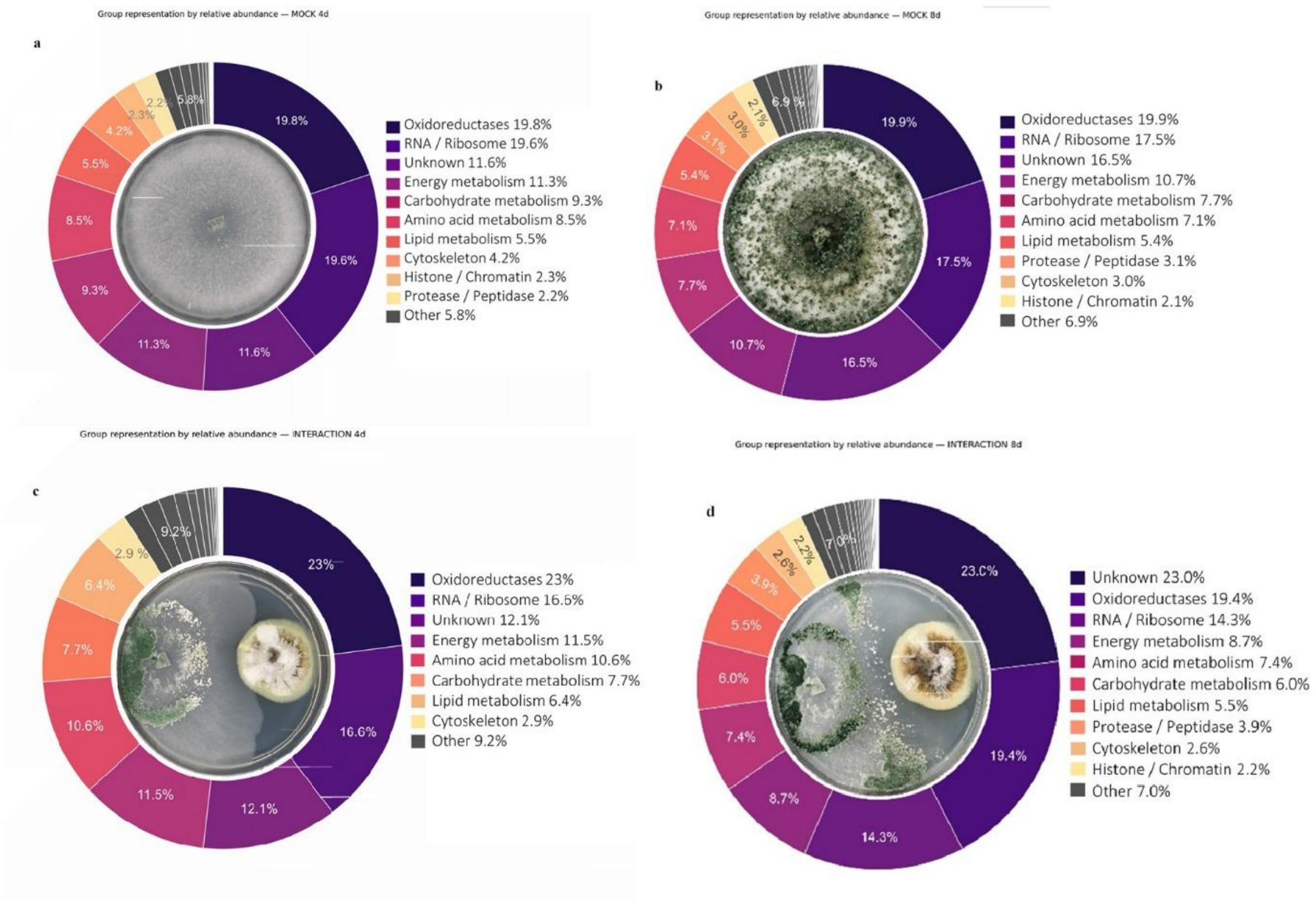

Figure S5: Functional composition of the *T. atroviride* proteome. Donut charts show category shares at (a) mock 4 dpi, (b) mock 8 dpi, (c) interaction 4 dpi, (d) interaction 8 dpi. Oxidoreductases dominate across conditions (~20 %); RNA/ribosome decreases from mock to interaction; the Unknown fraction rises at 8 dpi interaction.

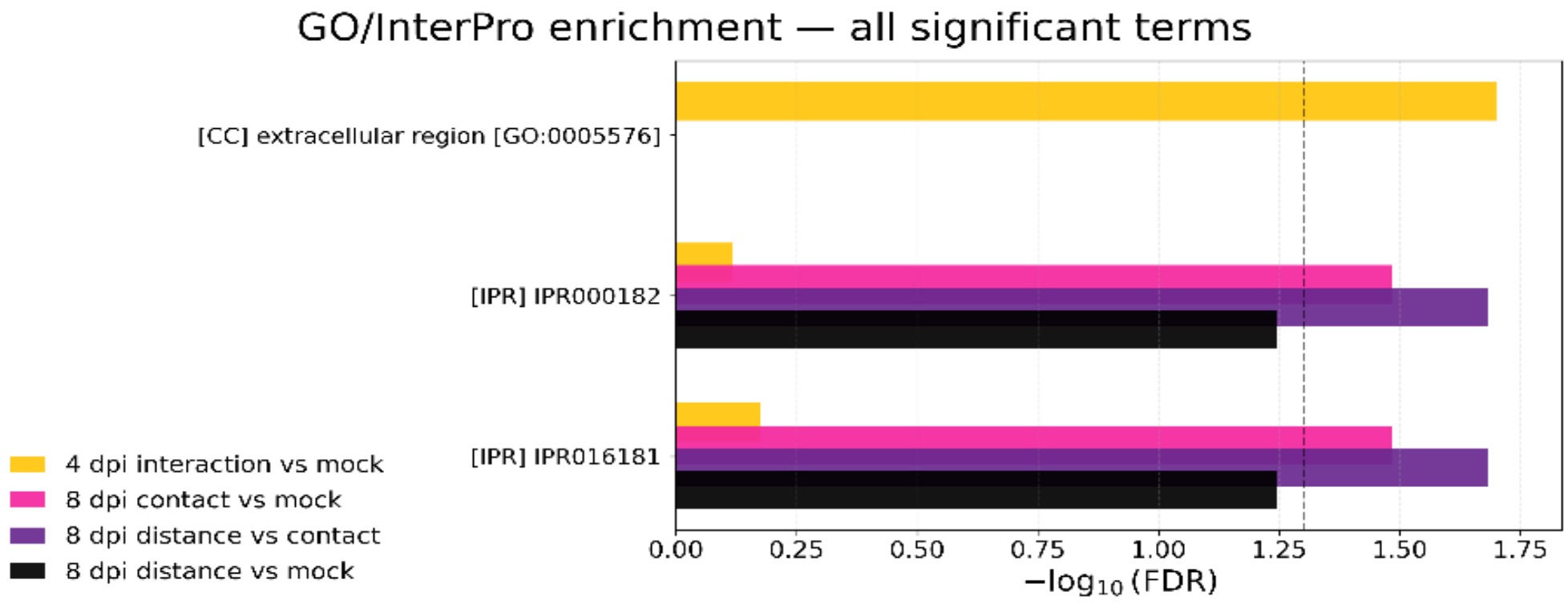

Figure S6: GO/InterPro enrichment and subcellular composition shifts. (a) Over representation analysis of upregulated proteins across four contrasts (4 dpi interaction vs mock; 8 dpi contact vs mock; 8 dpi distance vs contact; 8 dpi distance vs mock). Bars show $\log_{10}$(FDR); dashed line indicates FDR = 0.05. Significant terms include GO:0005576 (extracellular region) at 4 dpi and InterPro signatures IPR000182 and IPR016181 at 8 dpi (Supplementary Table S11). (b) Changes in compartmental contribution to the proteome (Δ percentage points) relative to the corresponding mock control for selected GO:CC terms (Tables S12–S13).

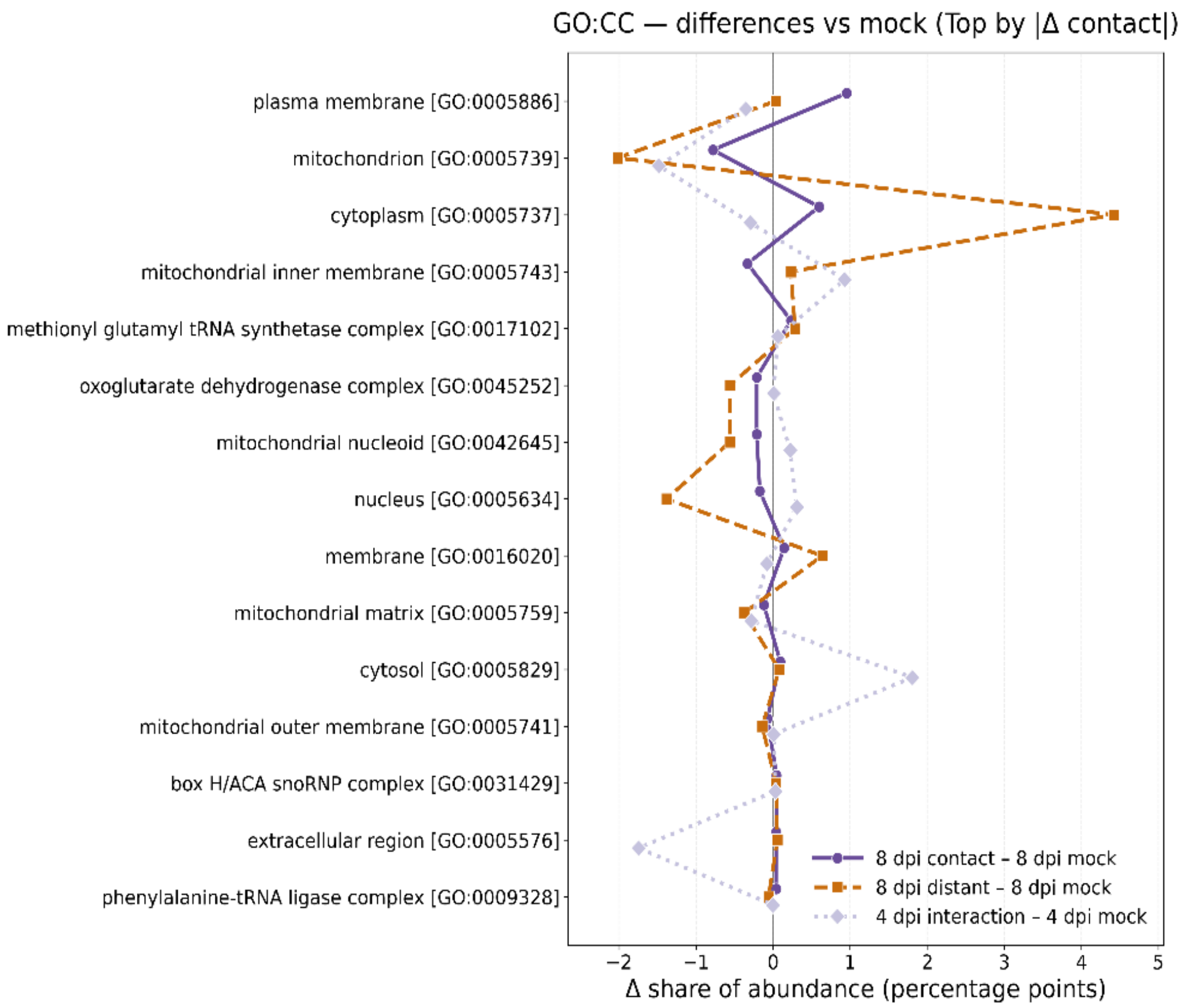

Figure S7: GO/InterPro enrichment and subcellular composition shifts. Changes in compartmental contribution to the proteome (Δ percentage points) relative to the corresponding mock control for selected GO:CC terms (Tables S12–S13).

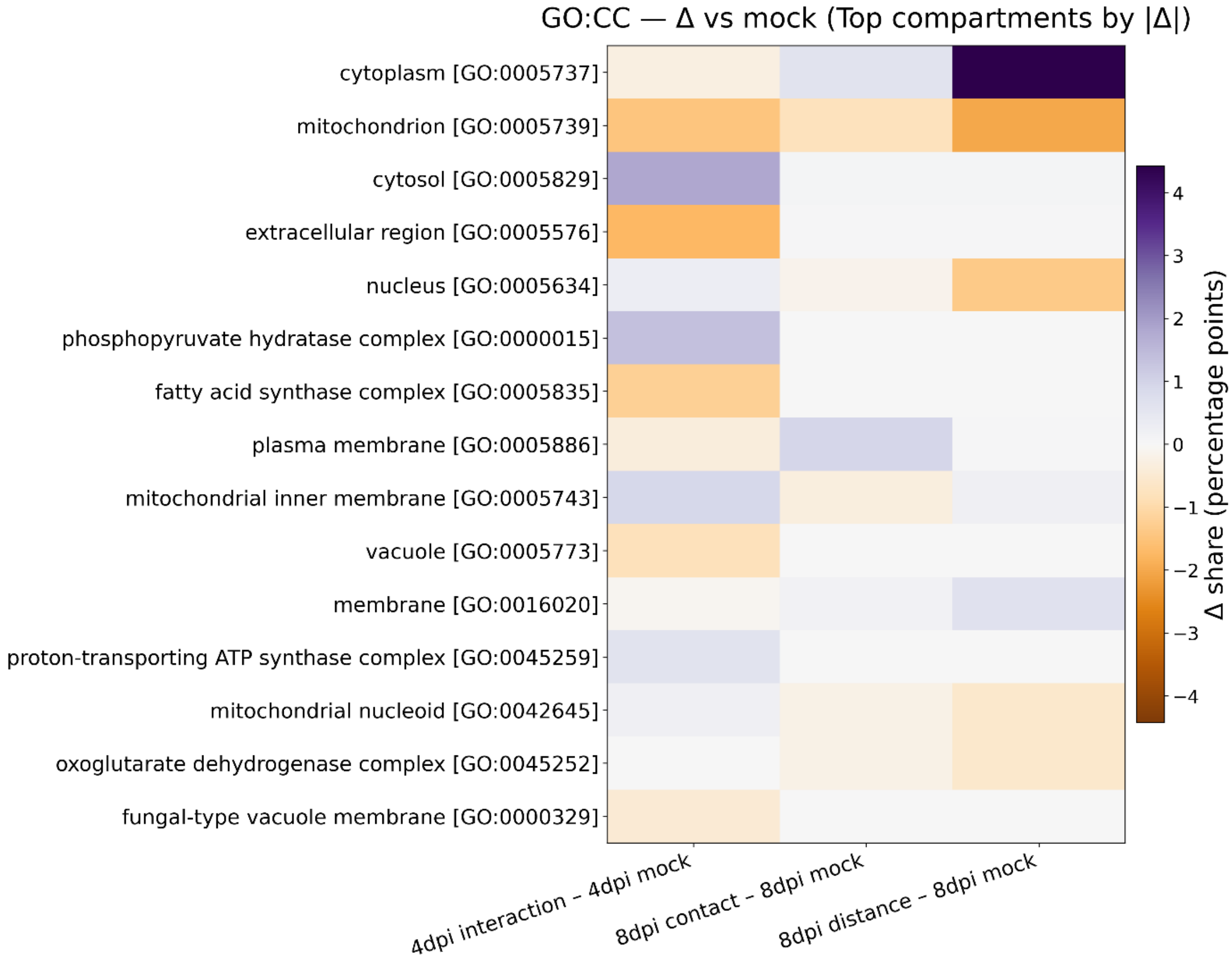

Figure S8: Enrichment and subcellular composition changes Heatmap of Δ shares for the cellular compartments with the largest absolute shifts (top compartments by max|Δ|) across all contrasts; zero denotes no change relative to the mock. Enrichment was tested using one-sided Fisher's exact tests with Benjamini–Hochberg FDR correction; the target set comprised proteins classified as up-regulated ($\log_2FC \geq 0.58$), and the background was restricted to GO-annotated proteins. Supplementary material Table S12, S13

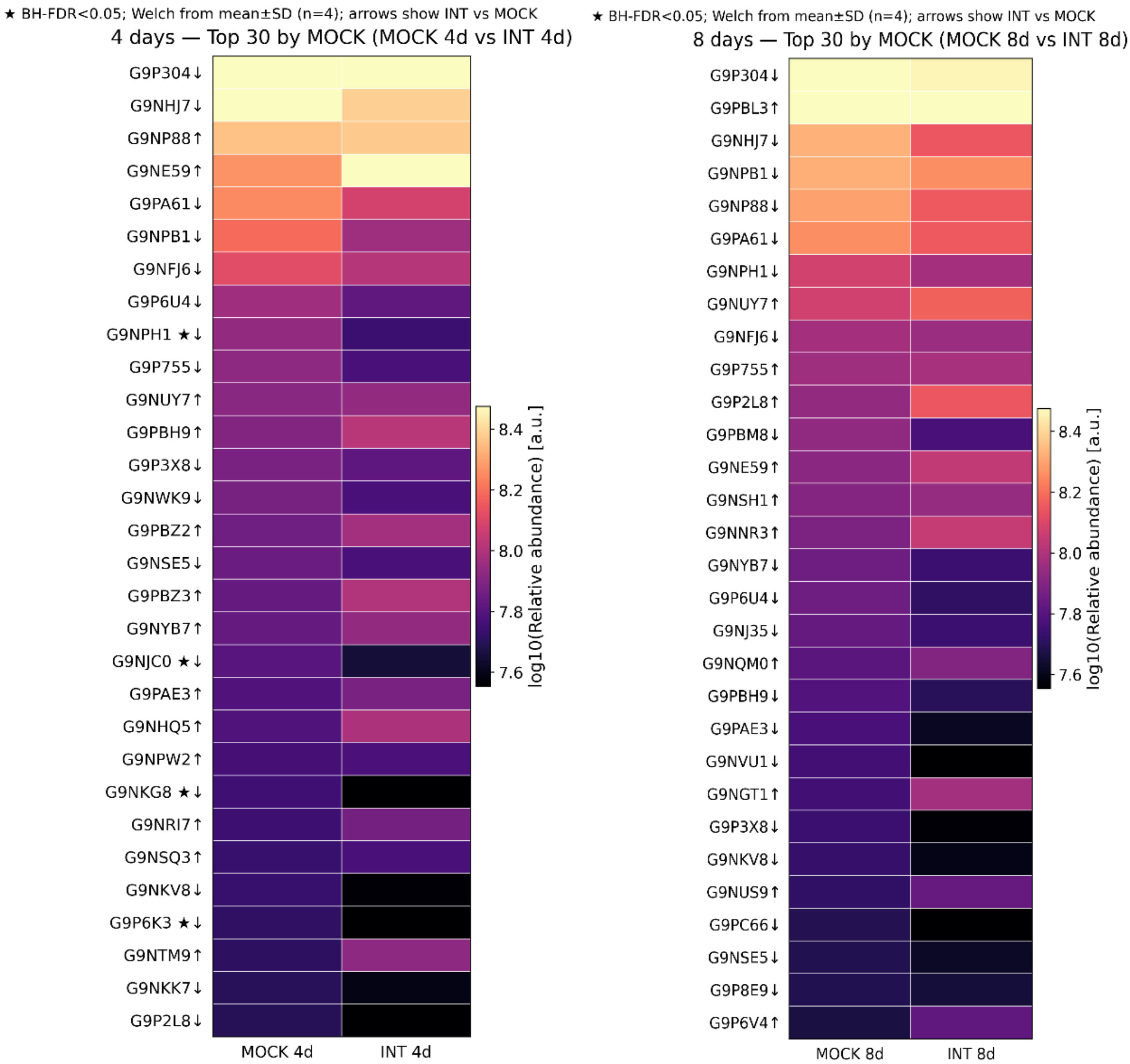

Figure S9: Contact re orders the top abundance proteome at 4 and 8 dpi. Heatmaps show the 30 proteins with the highest mean relative abundance in MOCK cultures and their abundance in the corresponding interaction (INT) samples (log_{10} relative abundance; mean of n = 4). Arrows indicate INT vs MOCK direction; ★ marks BH-FDR < 0.05 (Welch's t-test).

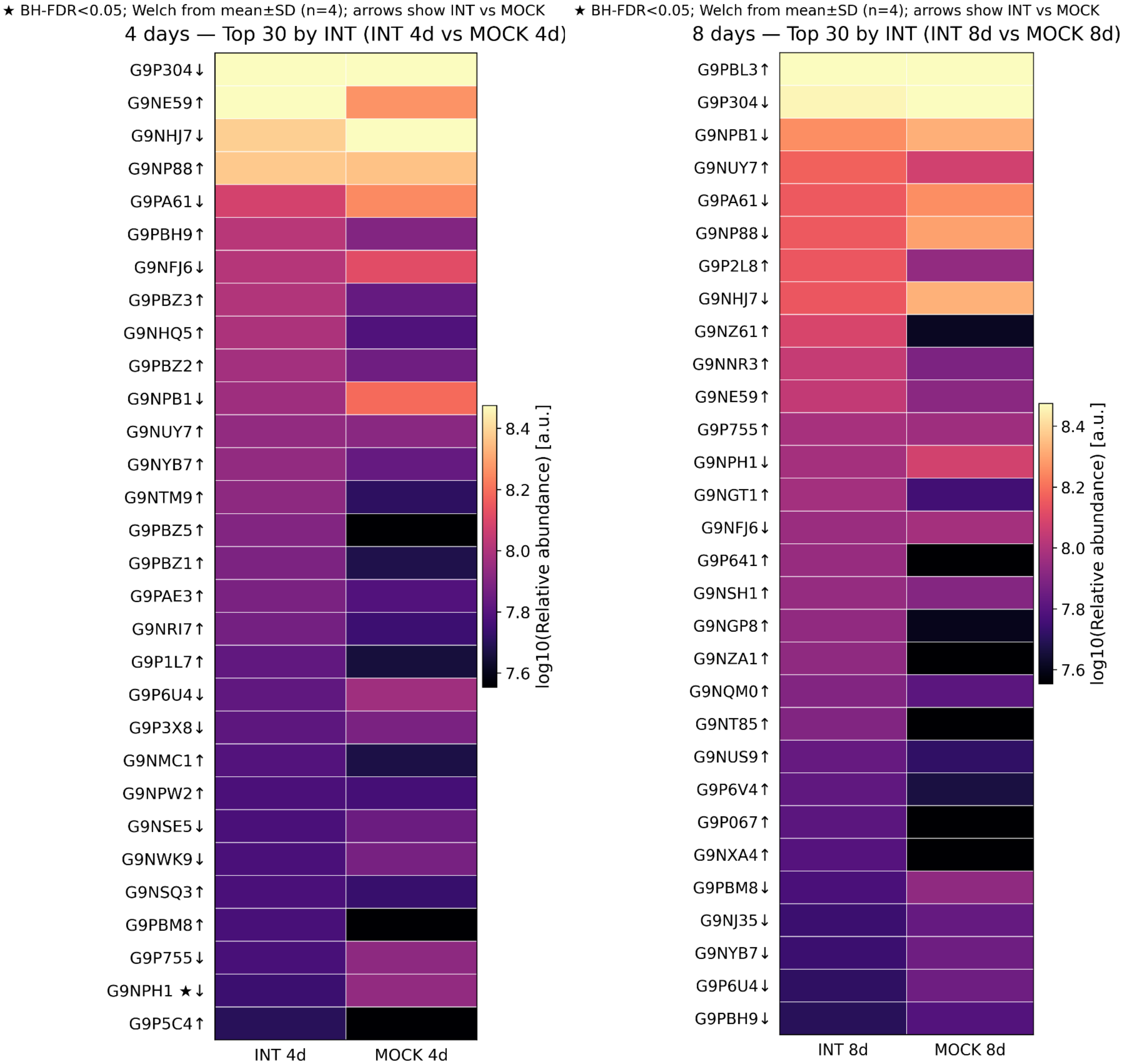

Figure S10: Reciprocal ranking (Top 30 by INT). Heatmaps show the 30 proteins with the highest mean relative abundance in INT cultures and their abundance in the corresponding MOCK samples (log_{10} relative abundance; mean of n = 4). Arrows indicate MOCK vs INT direction; ★ marks BH-FDR < 0.05 (Welch's t-test). Ranked lists are provided in Tables S16–S19.

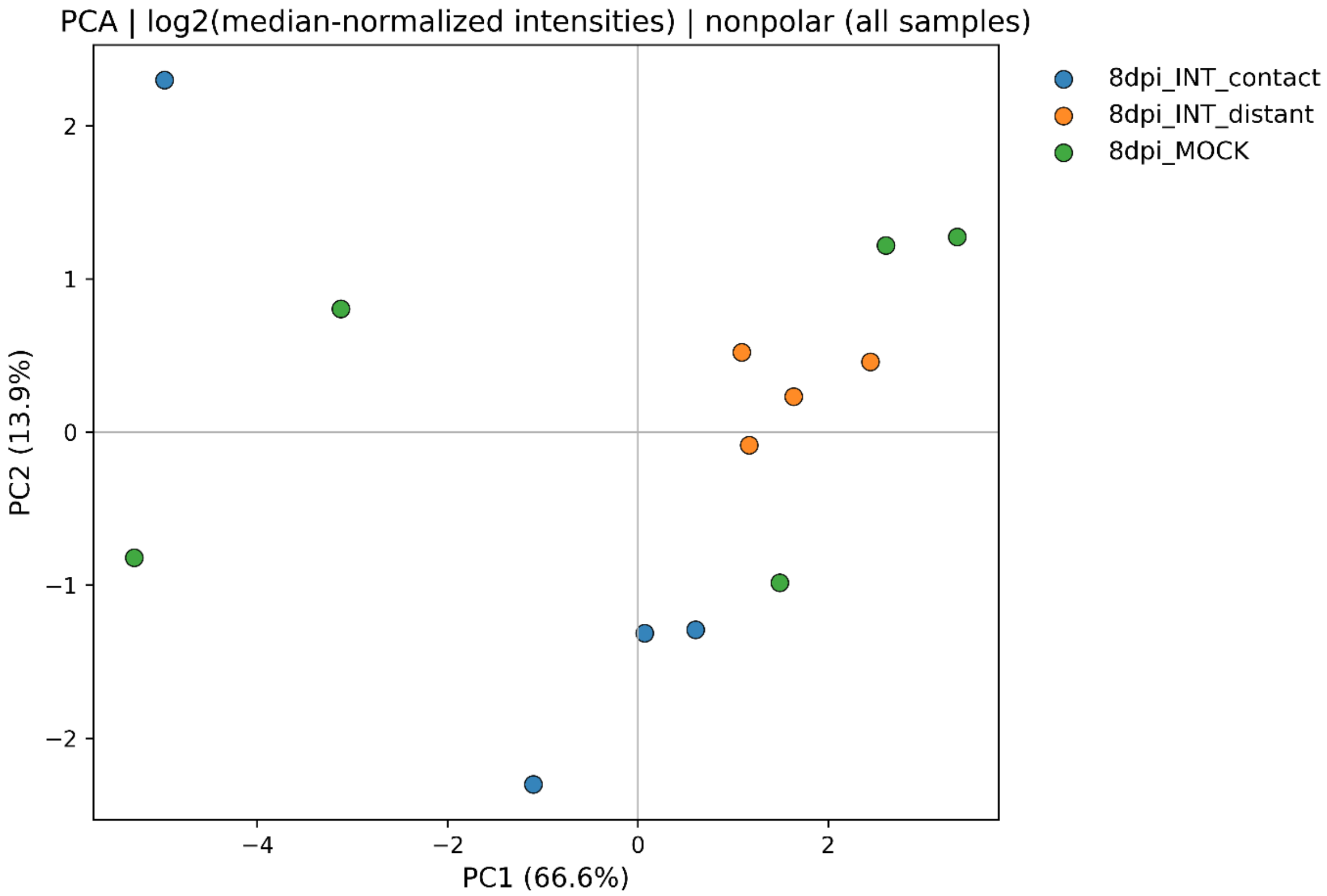

Figure S11: Nonpolar metabolome PCA. Principal component analysis (PCA) of nonpolar metabolite profiles based on log_2-transformed, median-normalized intensities. Points represent individual samples and are colored by condition (8 dpi INT_contact, 8 dpi INT_distant, 8 dpi MOCK). Axes show the variance explained by PC1 (66.6%) and PC2 (13.9%). PERMANOVA Table S 23.

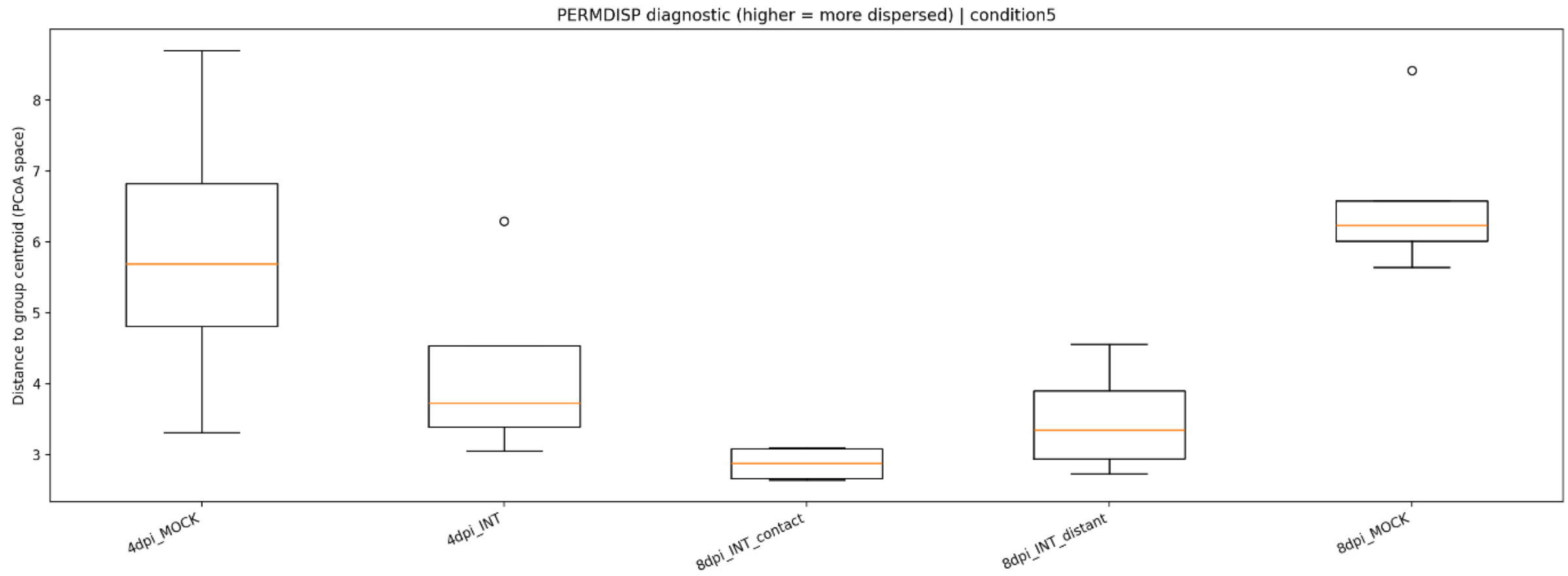

Figure S12: PERMDISP dispersion diagnostic across conditions. Boxplots show the distribution of sample distances to the group centroid in ordination space (higher values indicate greater within-group dispersion) for each condition (4dpi_MOCK, 4dpi_INT, 8dpi_INT_contact, 8dpi_INT_distant, 8dpi_MOCK) Table S22. Each point represents an individual sample; boxes indicate the interquartile range with the median, and whiskers show the data spread.

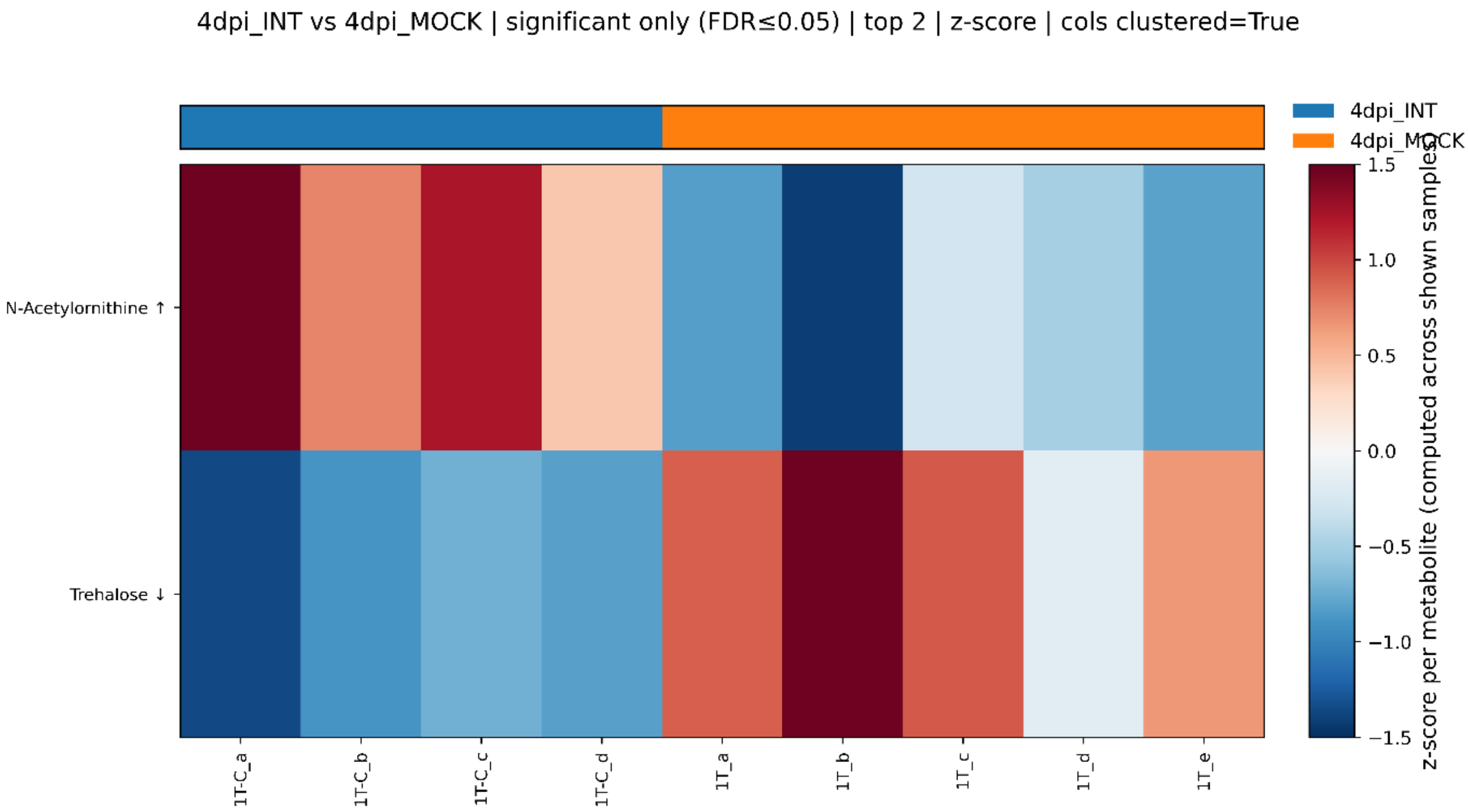

Figure S13: Differential polar metabolites at 4 dpi (INT vs MOCK) Table S27. Heatmap of the two significant metabolites (BH–FDR ≤ 0.05) for the 4dpi_INT vs 4dpi_MOCK contrast. Values are z-scores computed per metabolite across the samples shown (based on $\log_2$ median-normalized intensities). Columns represent individual replicates and are hierarchically clustered. N-acetylornithine is increased in INT (↑), whereas trehalose is decreased (↓) relative to MOCK.

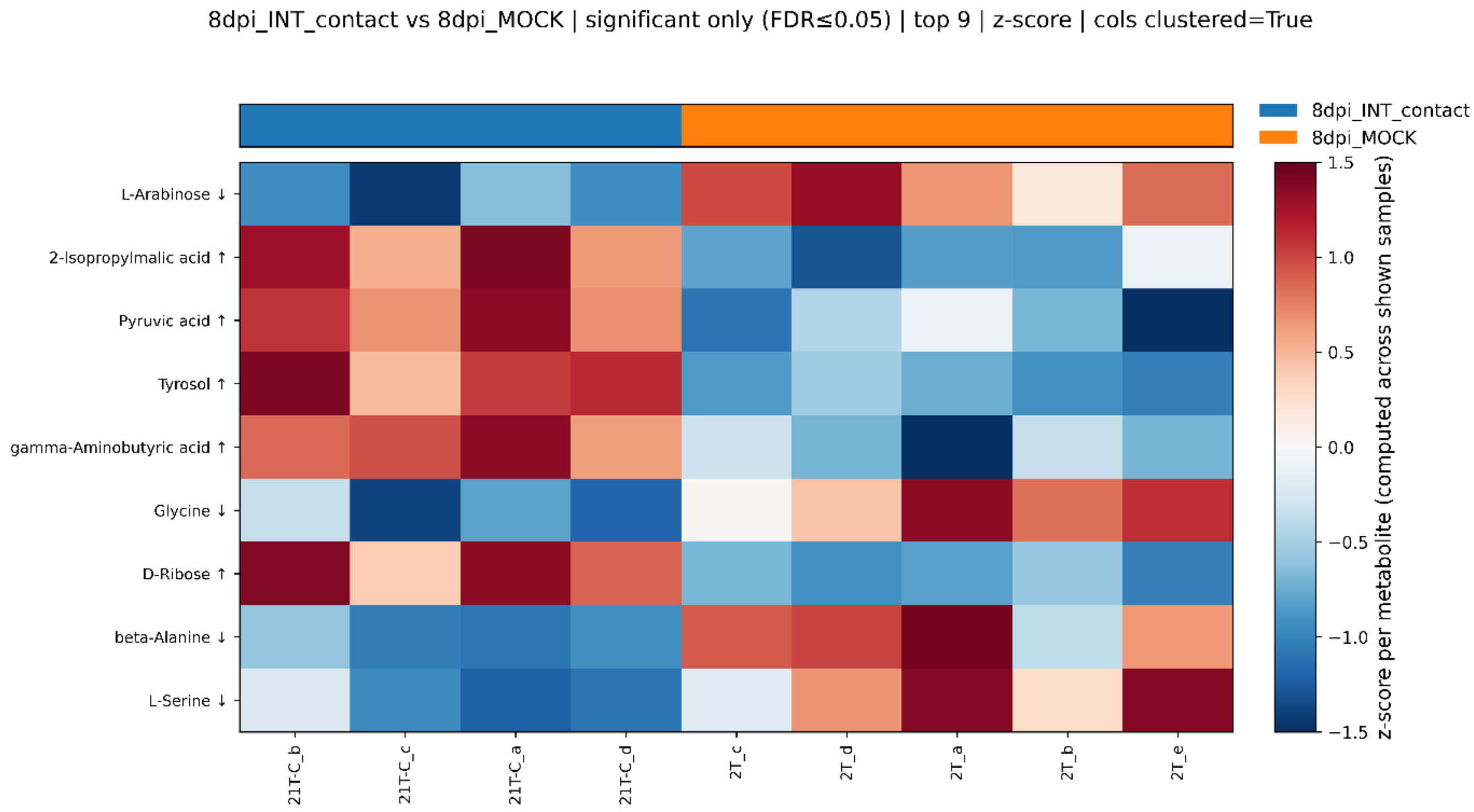

Figure S14: Differential polar metabolites at 8 dpi (INT_contact vs MOCK) Table S25. Heatmap of significant metabolites (BH–FDR ≤ 0.05) for the 8dpi_INT_contact vs 8dpi_MOCK contrast (shown: top 9). Values are z-scores computed per metabolite across the samples shown from $\log_2$ median-normalized intensities. Columns represent individual replicates and are hierarchically clustered. Metabolites with higher relative levels in INT_contact include tyrosol, pyruvic acid, 2-isopropylmalic acid, γ-aminobutyric acid (GABA), and D-ribose (↑), whereas L-arabinose, glycine, β-alanine, and L-serine are decreased relative to MOCK (↓).

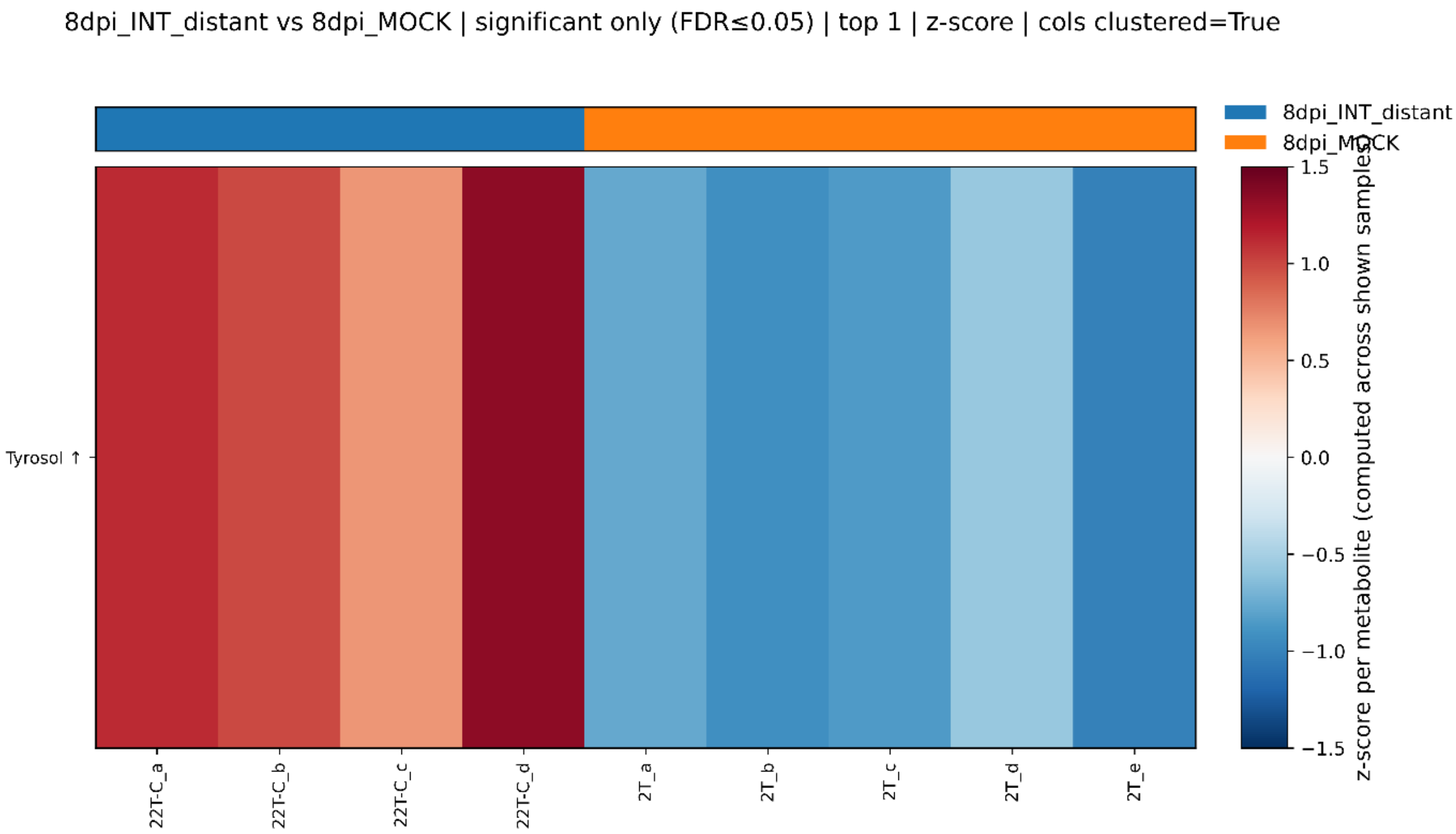

Figure S15: Differential polar metabolite at 8 dpi (INT_distant vs MOCK). Heatmap of the significant metabolite (BH–FDR ≤ 0.05; shown: top 1) for the 8dpi_INT_distant vs 8dpi_MOCK contrast Table S24. Values are z-scores computed per metabolite across the samples shown from log_2 median-normalized intensities. Columns represent individual replicates and are hierarchically clustered. Tyrosol is elevated in INT_distant relative to MOCK (↑).

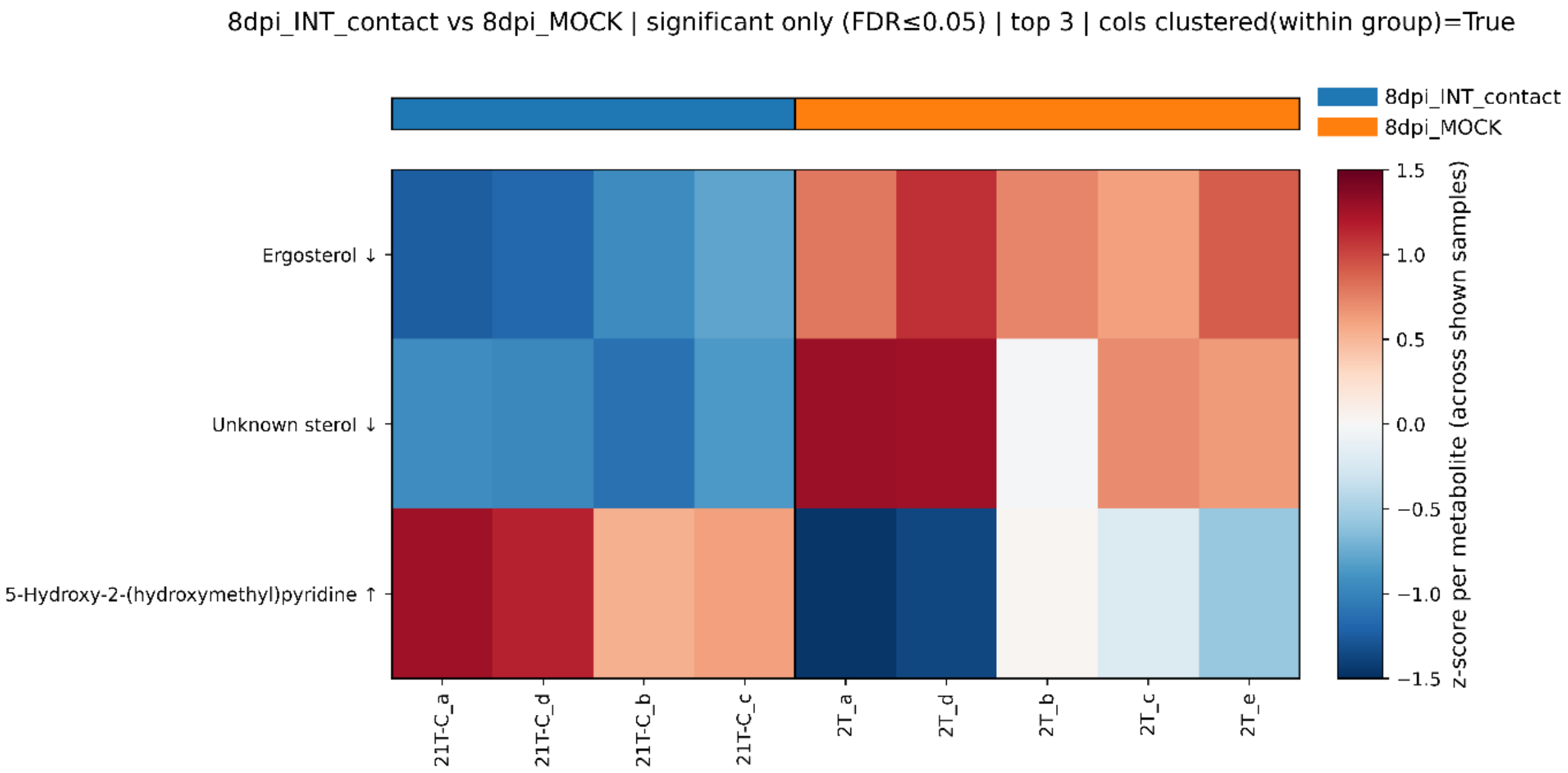

Figure S16: Nonpolar GC–MS heatmap of significant features in the contact zone versus mock at 8 dpi. Heatmap shows z-scored $\log_2$(median-normalized) intensities for the top three BH–FDR–significant nonpolar compounds (FDR ≤ 0.05) comparing 8 dpi INT_contact to 8 dpi MOCK, with columns clustered within groups. Ergosterol and an unknown sterol are decreased in the contact zone, whereas 5-hydroxy-2-(hydroxymethyl)pyridine is increased.
